# Non-random genetic alterations in the cyanobacterium Nostoc sp. exposed to space conditions

**DOI:** 10.1038/s41598-022-16789-w

**Published:** 2022-07-22

**Authors:** Yuguang Liu, Patricio Jeraldo, William Herbert, Samantha McDonough, Bruce Eckloff, Jean-Pierre de Vera, Charles Cockell, Thomas Leya, Mickael Baqué, Jin Jen, Dirk Schulze-Makuch, Marina Walther-Antonio

**Affiliations:** 1 Division of Surgical Research, Department of Surgery, Mayo Clinic, Rochester, MN 55905 USA; 2 Microbiome Program, Center for Individulized Medicine, Mayo Clinic, Rochester, MN 55905 USA; 3 Medical Genome Facility, Center for Individualized Medicine, Mayo Clinic, Rochester, MN USA; 4 Space Operations and Astronaut Training, Microgravity User Support Center (MUSC), German Aerospace Center (DLR), Linder Höhe, 51147 Köln, Germany; 5 School of Physics and Astronomy, University of Edinburgh, Edinburgh, EH9 3FD UK; 6 Fraunhofer Institute for Cell Therapy and Immunology, Branch Bioanalytics and Bioprocesses IZI-BB, 14476 Potsdam, Germany; 7 Astrobiological Laboratories, Planetary Laboratories Department, Institute of Planetary Research, German Aerospace Center (DLR), 12489 Berlin, Germany; 8 Astrobiology Group, Center of Astronomy and Astrophysics, Technische Universität Berlin, 10623 Berlin, Germany; 9 Section Geomicrobiology, GFZ German Research Center for Geosciences, Potsdam, Germany; 10 Department of Experimental Limnology, Leibniz-Institute of Freshwater Ecology and Inland Fisheries (IGB), Stechlin, Germany; 11 School of the Environment, Washington State University, Pullman, WA USA; 12 Department of Obstetrics and Gynecology, Mayo Clinic, Rochester, MN 55905 USA; 13 Department of Immunolgy, Mayo Clinic, Rochester, MN 55905 USA; 14 Mayo Clinic Graduate School of Biomedical Sciences, Mayo Clinic, Rochester, MN 55905 USA

**Keywords:** Bacterial genomics, Cellular microbiology

## Abstract

Understanding the impact of long-term exposure of microorganisms to space is critical in understanding how these exposures impact the evolution and adaptation of microbial life under space conditions. In this work we subjected *Nostoc* sp. CCCryo 231-06, a cyanobacterium capable of living under many different ecological conditions, and also surviving in extreme ones, to a 23-month stay at the International Space Station (the Biology and Mars Experiment, BIOMEX, on the EXPOSE-R2 platform) and returned it to Earth for single-cell genome analysis. We used microfluidic technology and single cell sequencing to identify the changes that occurred in the whole genome of single *Nostoc* cells. The variant profile showed that biofilm and photosystem associated loci were the most altered, with an increased variant rate of synonymous base pair substitutions. The cause(s) of these non-random alterations and their implications to the evolutionary potential of single bacterial cells under long-term cosmic exposure warrants further investigation.

## Introduction

Microorganisms with a high level of resistance to desiccation, ultraviolet (UV) radiation, vacuum tolerance^1^ and a capability of surviving in extreme environments such as cyanobacteria, lichen, fungi and green algae often serve as models for studies of the limits of terrestrial life^2^. For example, lichens have survived a 14-day low-Earth orbit flight in the BIOPAN-5 facility^3^; cyanobacteria and lichens have survived a 10-day space exposure in the Lithopanspermia Space Experiment^4^; and cyanobacteria, lichens and Antarctic fungi survived for 15 months on the EXPOSE-E and EXPOSE-R platforms on the International Space Station (ISS)^5^.

Among the aforementioned microorganisms, *Nostoc* is a genus of cyanobacteria growing on soil, rocks and pool substrates as well as a symbiont in other plants from the tropics to temperate and mountain climates on our earth, but also in extreme habitats from the frigid Antarctic valleys to hot desert soils^6^. Its survival is largely due to its ability to recover its metabolic activities within days after rehydration even after years of desiccation. Recently, *Nostoc* sp. CCCryo 231-06, a strain isolated from permafrost soil and rock substrates in Antarctica, was used for the 15-month Biology and Mars Experiment (BIOMEX) on the ISS^7^. Understanding the genomic evolution of this ecologically widely adapted species, also with regard to extreme environmental conditions, can serve as a guide for further studies and will be vital for a better understanding of how life adapts to space environments.

Single cell whole genome sequencing (SC-WGS) is being increasingly applied for investigating the genetic diversity and heterogeneity of complex biological systems^8^. However, environmental samples often consist of a biomass of complex cell communities and contaminants, making it difficult to identify genomic variants in low-abundance target cells^9^. With microfluidic technologies, it is feasible to isolate single cells from a population, and amplify femtograms of DNA for sequencing in a precisely controlled manner with minimal contamination^10^.

In this work, we performed microfluidic-based SC-WGS on single cells of the filamentous cyanobacterium *Nostoc* sp. CCCryo 231-06 exposed to space and Mars-simulated conditions for 23 months on the ISS. Experimental conditions included UV and cosmic radiation exposure, microgravity, and the use of lunar and martian analog soils on the ISS and in a Mars simulation chamber on Earth, to better understand the impact of these parameters. Our goal was to provide insights into genomic changes at the cellular level experienced by a species able to survive long-term exposure to space conditions.

## Materials and methods

### Sample preparation

**Figure 1:**
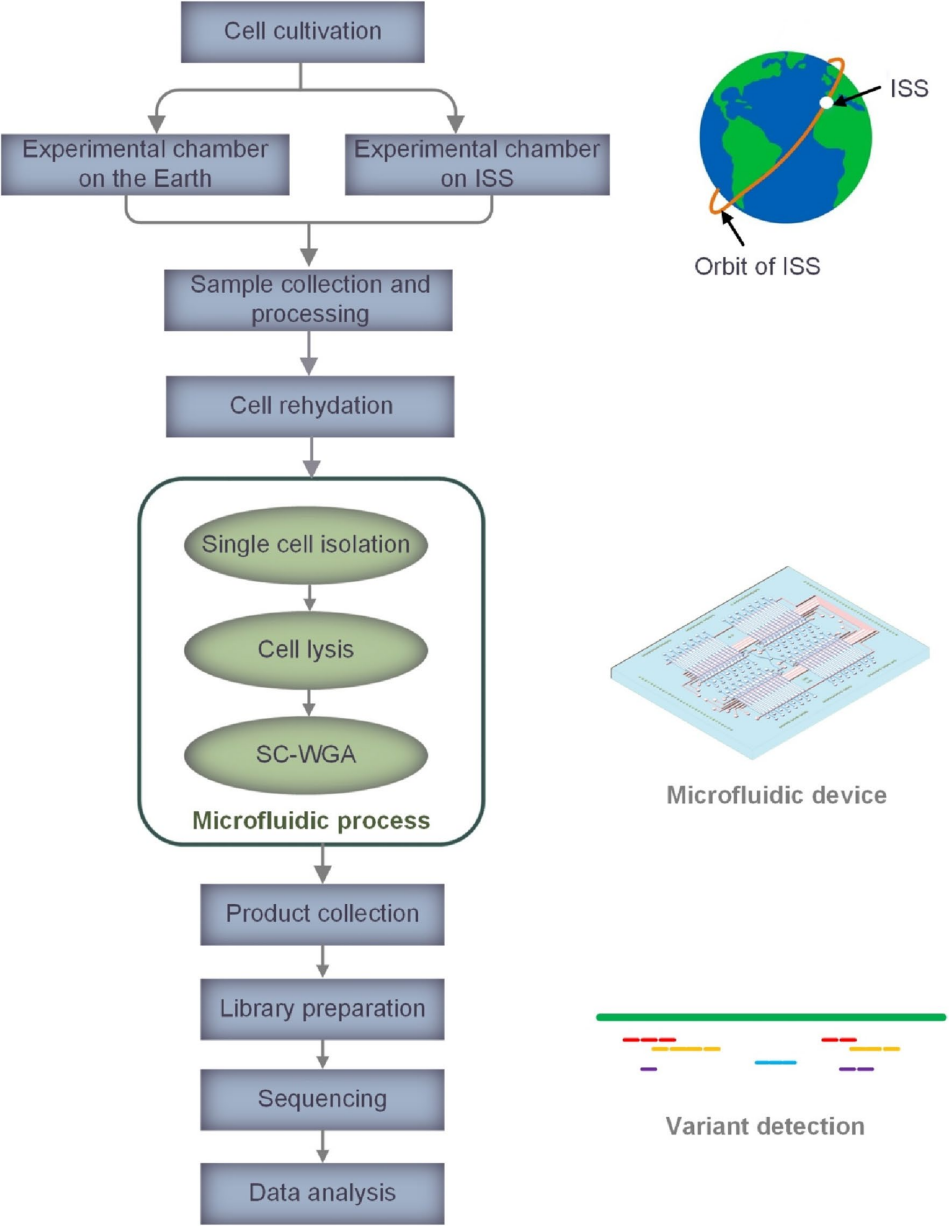
An overview of the workflow of SC-WGS for detecting variants in the *Nostoc* sp. strain CCCryo 231-06.

The Antarctic strain CCCryo 231-06 (= UTEX EE21; CCMEE 391) of the cyanobacterium *Nostoc* sp*.* (hereafter, for readability addressed as "the *Nostoc*" only) was used as the target strain in this work. Briefly, the sample was prepared pre-flight, and the samples were on the ISS for 23 months, placed outside the ISS for 17 months, and exposed to UV for 15 months. These samples were returned to Earth and collected for post-flight processing before SC-WGA in a microfluidic device for sequencing and analysis. The overall workflow is illustrated in Fig. 1. Details of strain origin, pre-flight sample preparation, BIOMEX experimental parameters and post-flight sample preparation can be found in the supplemental information (SI) S1.

### Single cell sequencing

#### Microfluidic experimental setup

**Figure 2:**
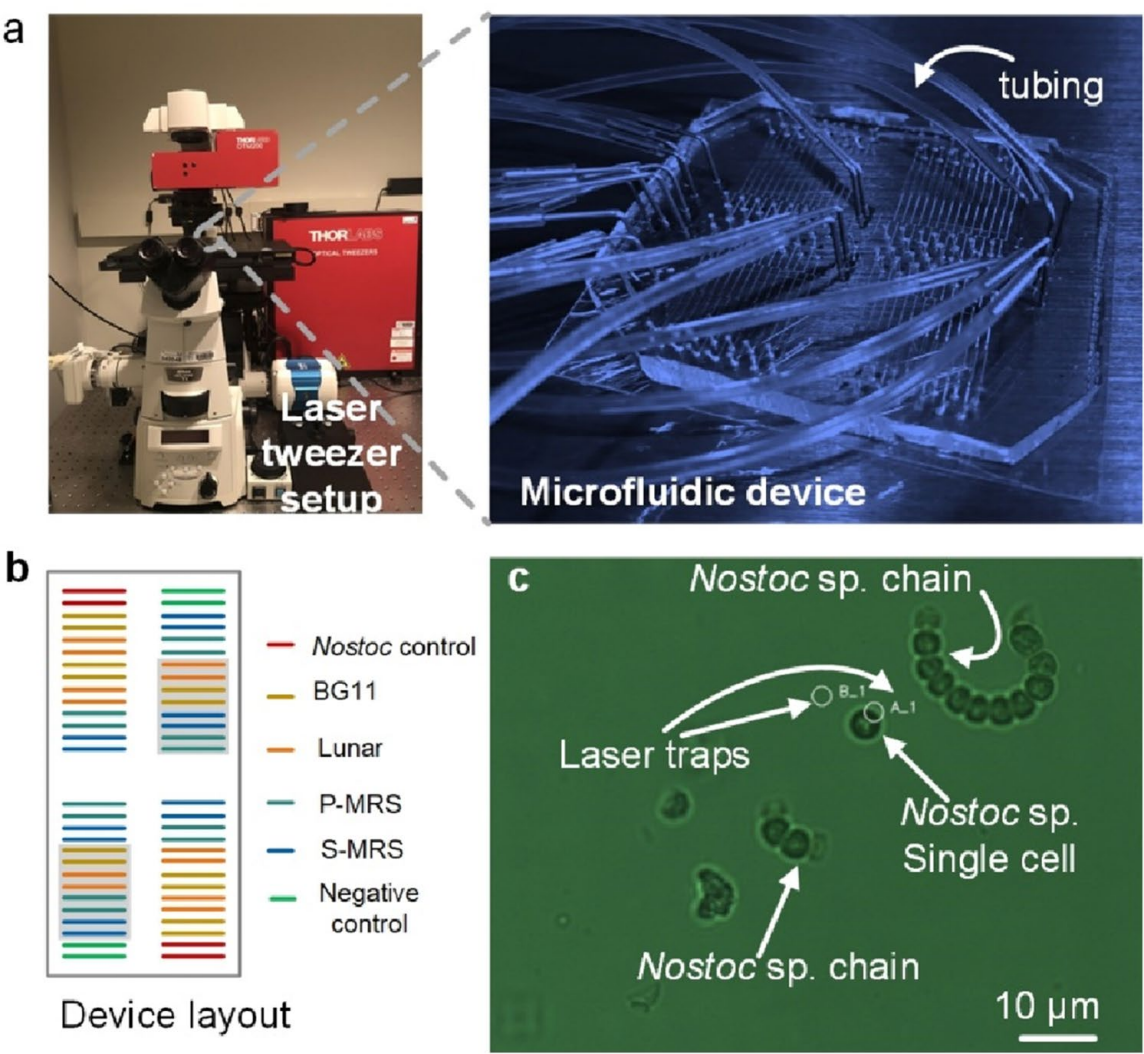
Optofluidic platform overview. (**a**) An optofluidic platform consists of a microscope, laser tweezers and a microfluidic device connected with tubings for high-throughput SC-WGA. (**b**) A schematic diagram of the microfluidic device with 4 identical reaction blocks with 14 reaction lines in each block. The location of samples and controls are shown as color coded. Experimental ground samples are indicated by the grey shades. (**c**) The use of laser tweezers to trap *Nostoc* CCCryo 231-06 single cells. Laser trap A_1 was turned on and trapped a single cell in the sample suspension. Laser trap B_1 was turned off. *S-MRS* sulfatic martian regolith, *P-MRS* phyllosilicatic martian regolith.

SC-WGA was performed in an optofluidic platform at Mayo Clinic (Rochester, MN)^11–13^ with a customized microfluidic device for high-throughput SC-WGA. Briefly, this platform consists of a microscope (Nikon Eclipse), optical tweezers (Thorlabs) and a custom-built Polydimethylsiloxane (PDMS) microfluidic device with 4 identical reaction blocks that contain 14 parallel reaction lines in each block (Fig. 2a). Each reaction line has sets of valves that allow for the creation of isolated microchambers. The number of microchambers in each reaction line corresponds to the number of reagents that needs to be sequentially added to perform the SC-WGA reactions. The sample inlets of the microfluidic devices were designed to support the injection and isolation of cells under different experimental conditions in an individual manner, therefore minimizing cross-contamination between samples.

Each reaction block in the microfluidic device was designed to accommodate 13 single cells from the filaments of the *Nostoc* exposed to different conditions as listed in Table S1 and one negative control (PBS). Our original plan was to perform SC-WGA on 4 single cells from each of the 13 samples, however, it was increasingly difficult to identify *Nostoc* cells in sample No. 7 and sample No. 13, and thus, only three single cells and one single cell from these samples were recovered and sequenced (Table S1) respectively.

#### Microfluidic-based SC-WGA

The desiccated samples were rehydrated according to the procedure described in the supplemental information S1. Prior to introducing the cells into the microfluidic device, the sample channel in the microfluidic device was primed with chip diluent (0.04% Pluronic F127 in PBS) for 30 min to prevent the cells from sticking to the PDMS surface. The prepared cell suspension was then injected into the device. To reduce possible location-induced sequencing bias when performing SC-WGA in a microfluidic device, each sample was injected through channels at two different locations in the device, with two cells of each sample isolated into adjacent reaction lines at each location. The sample placement scheme is shown in Fig. 2b. The shaded areas represent samples from the ground simulation experiments.

Single cells were trapped and transported into microchambers by optical tweezers. Visually identifiable contaminating cells were trapped and moved out of the chambers to ensure only the target cell was in the chamber prior to the lysis step. The major advantages of using optical tweezers to isolate single cells from a population include high target single cell confidence, providing a way to visually ensure that only one cell is trapped into a microchamber and thus maintaining minimal possibility of sequencing contaminating cells unintendedly. A representative image of using the laser tweezers to trap a single *Nostoc* cell is shown in Fig. 2c. Lysis of the *Nostoc* cells and genome amplification procedure followed our optimized protocol for bacterial single cell lysis and whole genome amplification in microfluidic devices for downstream sequencing^11^. The protocols for *Nostoc* lysis and library construction and sequencing can be found in SI.

### Bioinformatic data analysis

#### De novo genome assembly

The sequenced reads were pre-processed prior to de novo genome assembly (see SI). One of the major challenges of bacterial SC-WGS is that the whole genome of a target single cell can only be partially recovered. However, with sufficient number of single cells (regardless of different experimental conditions), it is possible to co-assemble a consensus *Nostoc* genome to near completion. Therefore, in this work, we co-assembled using all 48 samples targeting the *Nostoc* isolate^14^. We emphasize the “consensus” aspect of this recovered reference sequence, and consequently this sequence will not completely match the genotypes of samples from each single exposure condition. This co-assembled consensus genome was to be used as a reference for the variant calling procedures and as a template for gene/function annotation to inform the significance of the detected variants. In this case, we determined that the consensus reference would be an acceptable substitute for the purpose of identifying variants of interest and offering insights into the functions present in *Nostoc* sp. CCCryo 231-06.

Specifically, we combined the reads from all 48 samples, and to offset the uneven coverage of sequenced reads introduced by the whole-genome amplification process, we digitally normalized the read coverage using the BBNorm tool from the BBTools suite version 38.26^15^ to a target coverage of 100X. We then took the combined, digitally-normalized reads and used the MEGAHIT de novo metagenomic assembler version 1.1.3^16^ using the “meta-sensitive” preset.

To reconstruct the consensus *Nostoc* sp. genome, we needed to separate its contigs from the contaminating contigs from other organisms (non-cyanobacterial bacteria, fungi). We used the BusyBee tool to identify and select the bin of our target organism^17^. We then assessed the initial quality of the recovered genome including the completeness and contamination based on a set of normally single copy gene markers using checkM version 1.0.13^18^, and then refined the bin using the refine tool following the procedure outlined in Parks, et al.^19^. Finally, we used the GTDBtk tool version 0.2.2^20^ to putatively determine the taxonomic placement of the recovered genome. The genome was annotated using the RASTtk pipeline through the PATRIC service^21^. Details of metagenomics analysis and base quality score recalibration are described in SI.

#### Variant calling

We used the recovered genome of the *Nostoc* sp. CCCryo 231-06 as a reference for variant calling. For each of the *Nostoc* samples and replicates, we mapped the reads with recalibrated base quality scores against this reference using BBMap, then coordinate-sorted these reads using SAMtools. The variants were called using GATK4 version 4.0.1.0 HaplotypeCaller in haploid mode^22^. We used SnpEff version 4.3t to annotate variants and predict effect, and SnpSift version 4.3t to filter low quality variants^23^. To remove the spurious variants resulting from the amplification procedure, we merged the replicates for each sample using BCFtools, requiring that each putative variant is present in at least two replicates. This way, most of the spurious variants will be removed. Finally, the variants were visualized using the web version of Integrative Genome Viewer.

#### Variant analysis

**Figure 3:**
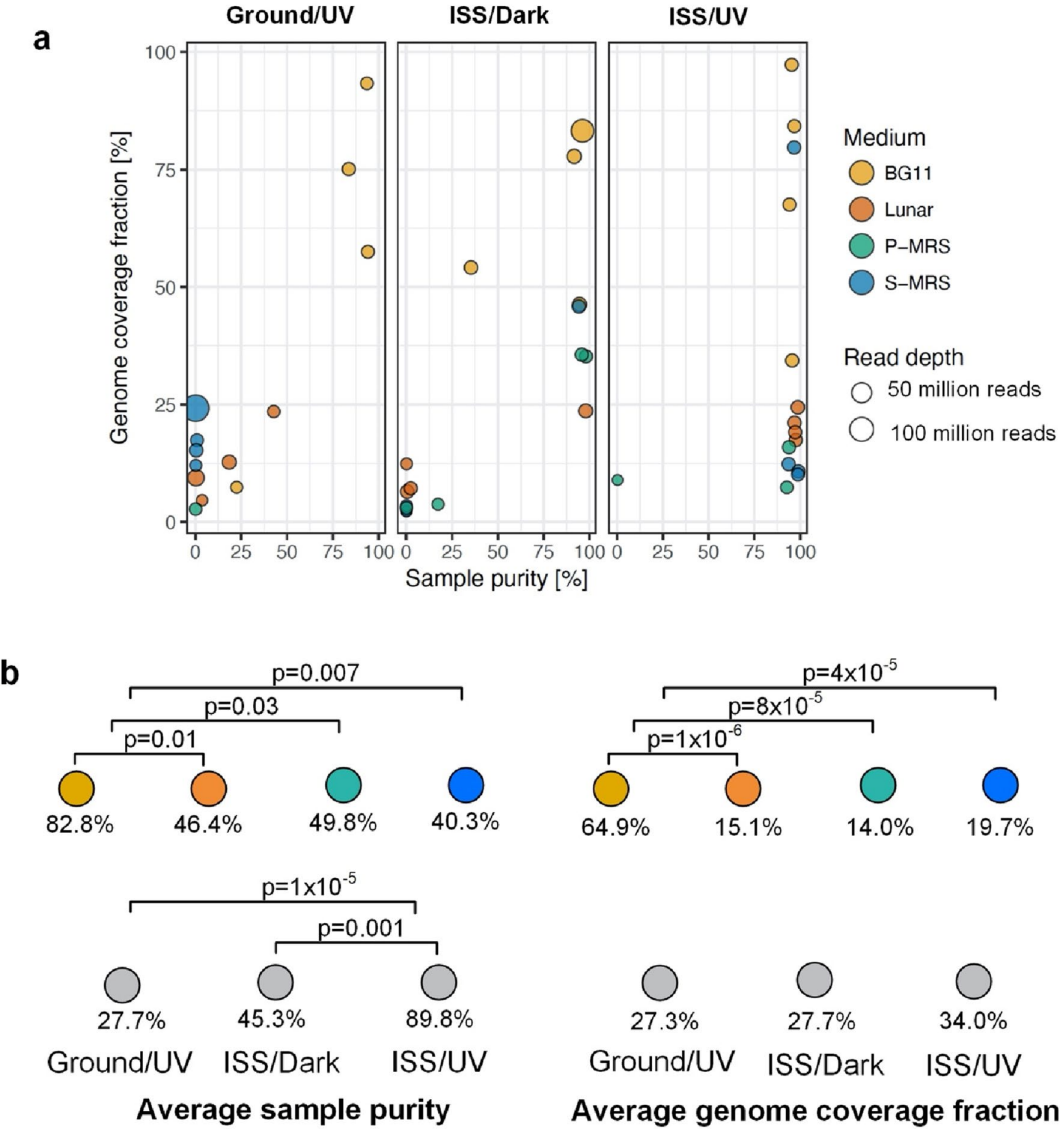
Sample purity and genome coverage of single cells of *Nostoc* sp. CCCryo 231-06 exposed to different media/substrates and exposure conditions during the BIOMEX experiment. Number of replicates per experimental conditions are described in Table S1. Different media/substrates are color-coded. (**a**) The horizontal axis represents the percentage of reads that were classified to the genus *Nostoc* after the metagenomic analysis of each sample and replicate. The vertical axis represents the fraction of the recovered *Nostoc* sp. CCCryo 231-06 that is mapped by the reads of each sample and replicate. The size of the circle represents the number of reads present in each sample and replicate. The components of the mineral substrates (lunar and Martian regoliths) are provided in Table S2. (**b**) Average sample purity and genome coverage fraction are sorted by medium and exposure condition. Samples on BG11 displayed significantly higher purity and genome coverage. Samples exposed to UV radiation on the ISS displayed significantly higher purity than those in other experimental conditions. Student’s t test was used to calculate the p-values. *BG11* blue-green medium, *ISS* international space station, *P-MRS* phyllosilicatic martian regolith, *S-MRS* sulfatic martian regolith, *UV* ultraviolet light, *Ground/UV* earth with UV exposure, *ISS/Dark* ISS without UV exposure, *ISS/UV* ISS with UV exposure.

Whole genome and variant gene breadth of coverage were calculated using SAMtools. Variant analysis was performed on ISS/UV, ISS/Dark, and Ground/UV samples only as the gene coverage for samples grown on Mars and Lunar media was too poor (Fig. 3a). Synonymous to non-synonymous variant ratios (Ka/Ks ratio) were calculated by counting the respective variants in each replicate, gene-by-gene, and normalized according to the breadth of coverage of the area of interest^24^. Genes were selected to be significant with Ka/Ks ratios greater than 1, indicating non-synonymous alterations or with Ka/Ks ratios less than 1, indicating synonymous alterations. Genes that met these criteria through at least two experimental conditions were analyzed for significance across replicates and within the genome with the Friedman test and Nemenyi post-hoc test. Probabilities of variant distributions on a gene-by-gene basis were calculated using Pascal’s formula where N = length of gene and K = number of observed shared variants between conditions. Probabilities of genome-wide variant distribution were tested using an algorithm in which the whole genome was treated as a continuum. To assess whether the observed variants could be attributed to a random distribution, one million iterations of an equivalent set of simulated variants were created and randomly assigned to a location in the genome.

#### Protein structure analysis

The consensus amino acid sequence of the *Nostoc*’s photosystem II D1 protein PsbA, obtained for the Ground/UV, ISS/UV, ISS/Dark experimental conditions, as well as for the reference genome, was submitted to the RaptorX Structure Prediction server to predict its tertiary structure^25^. Each predicted structure was returned in PDB format, and they were structurally pairwise-aligned using PyMOL v2.3.0^26^. PyMOL was then used to visualize the effects of variants across experimental conditions by showing structural differences and non-synonymous variants in the context of its tertiary structure. The nucleotide and amino acid sequences of proteins of interest were visualized using the method described in SI.

#### Contamination

Organisms in the samples that did not belong to the *Nostocaceae* were counted as contaminants. Sequenced single cells from space and ground samples on BG11 medium displayed an average of 82.8% sample purity, significantly higher than the purity of single cells on Lunar (46.4%), P-MRS (49.8%) and S-MRS (40.3%) regolith analogs with all p-values < 0.05. As the regolith analogs are composed of multiple mineral components collected from various locations, there is a higher possibility of the mineral mixtures carrying contaminants. However, these minerals were autoclaved prior to plating the microorganism, thus it is more likely that the contaminants were introduced during sample transfer and handling. In either case, we assumed that contaminants did not influence the experimental results on the ISS and respective variants.

## Results

### Sample purity and genome coverage

To investigate possible changes in the genotype of *Nostoc* sp. CCCryo 231-06 under space conditions, we calculated sample purity and genome coverage of single cells exposed to different experimental conditions. The normalized sample purity and genome coverage of single cells of *Nostoc* sp. CCCryo 231-06 is shown in Fig. 3a. For samples on the ISS, the purity of those that were UV-exposed was ~ 89.8% (SD = ± 24.8%), approximately twice as pure as samples without UV exposure (p ≤ 0.03, Student’s t-test). This is in agreement with other studies demonstrating that UV radiation often leads to structural and genetic damage in various companion microorganisms in non-axenic cultures^27^, leading to their lower survivability under extreme conditions. However, although the UV intensity in the simulation chamber on Earth was approximately twice that on the ISS, samples that remained on Earth were only < 33% as pure as those that were exposed to UV on the ISS (Fig. 3b). This raises the possibility that the combination of cosmic and UV radiations poses significant challenges to the survivability of most accompanying microorganisms. Further enhanced purity was observed under growth conditions on Blue-Green culture medium (BG11) relative to desiccated conditions on lunar or Mars-analog regolith (p ≤ 0.03, Student’s t-test). BG11 is one culture medium of several that is tailored to support growth of cyanobacteria like *Nostoc*, and therefore is likely to provide a competitive advantage over companion microbes. Samples of strain CCCryo 231-06 on the BG11 medium resulted in significantly higher genome coverage than those on lunar or Martian analogs. Specifically, the single cells on BG11 showed an average of 64.9% (SD = ± 26.3%) genome coverage, while in the cells on other substrates < 20% coverage was achieved (p ≤ 8 × 10^–5^, Student’s t-test) (Fig. 3b). While BG11 is widely used for cyanobacterial cultures and is well characterized and used in molecular assays, that is not the case for the lunar and Martian-analog substrates. It is possible that the latter contain compounds that have inhibitory effects on molecular reactions, interfering with the effectiveness of lysis and genomic amplification procedures. No statistically significant differences between the genome coverage of samples exposed on the ISS and on Earth were observed.

### Genomic variant comparison

**Figure 4:**
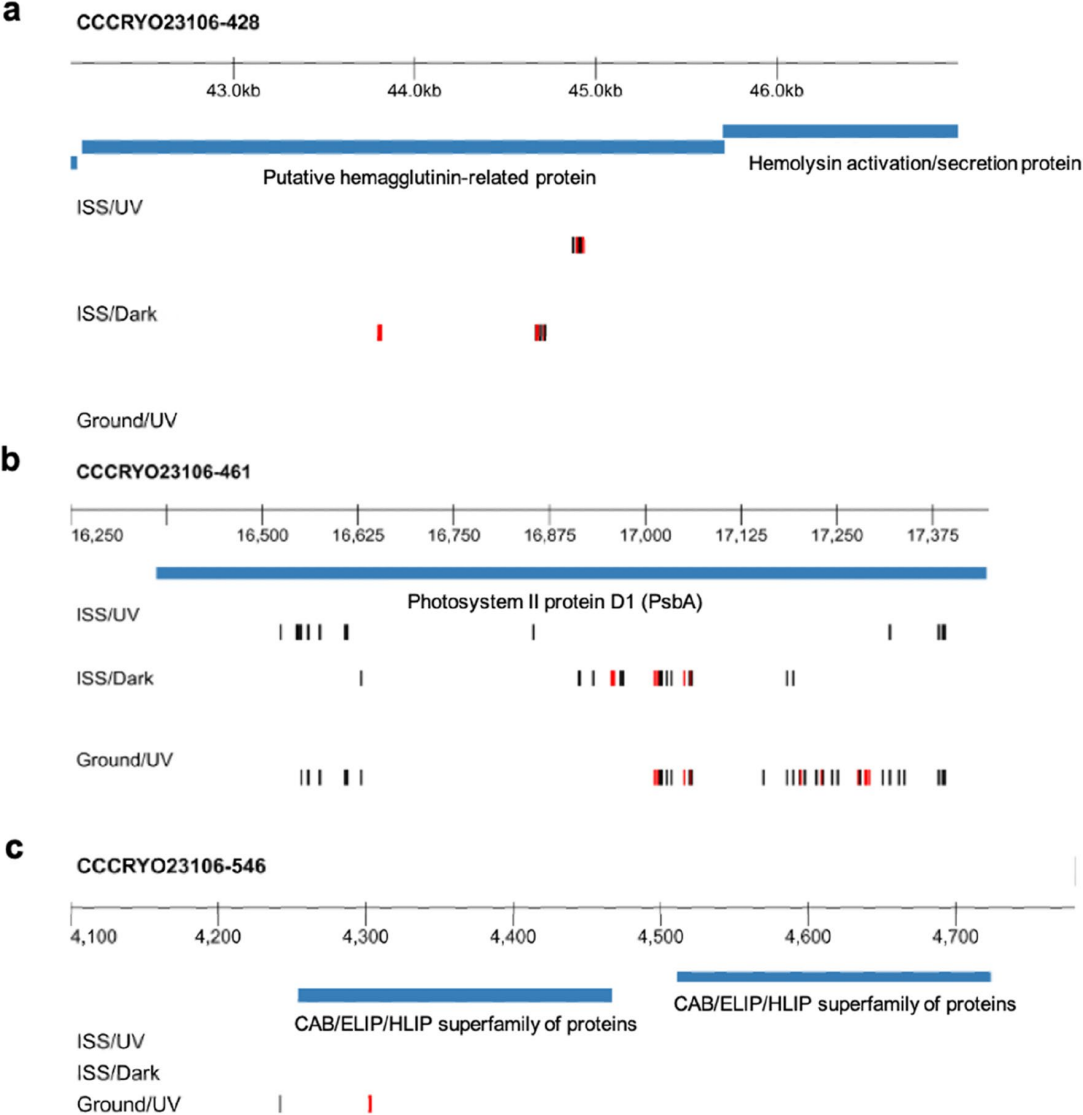
Plot of variants on BG11 medium detected in biofilm-associated hemagglutinin gene and photosynthesis-associated genes. (**a**) Biofilm-associated hemagglutinin genes. (**b**) Photosystem II D1 (*psbA*) gene. (**c**) High-light inducible protein genes. Each track represents a medium/exposure condition, and the blue markers represent variant positions with respect to the consensus reference genome for *Nostoc* sp. CCCryo 231-06. Black vertical lines represent synonymous variants (not impacting amino acid translation), red vertical lines represent non-synonymous variants (impacting amino acid translation). *ISS* international space station, *UV* ultraviolet light, *ISS/UV* ISS with UV exposure, *ISS/Dark* ISS without UV exposure, *Ground/UV* earth with UV exposure.

We used the recovered genome of the *Nostoc* sp. CCCryo 231-06 (NCBI BioProject accession number PRJNA721463; Whole Genome Shotgun project has been deposited at DDBJ/ENA/GenBank under the accession JAHCSU010000000; genome announcement submitted elsewhere) as a reference for variant calling. To minimize artifacts or errors, we required that the same exact genomic variant be present in at least two single cell replicates exposed to the same condition to be called a variant. Genomic variants were only identified in samples on BG11, which may be a result of the low genomic coverage for the other substrates utilized (Fig. 3a). Therefore, variant analysis was performed on ISS/UV (ISS with UV exposure), ISS/Dark (ISS without UV exposure), and Ground/UV (Earth with UV exposure) samples on the BG11 medium only. We identified genomic variants in genes involved in biofilm production and/or photosynthesis. Biofilm associated variants appeared in hemagglutinin-related genes in samples from the ISS (in both UV and dark conditions) (Fig. 4a). While genomic variants related to photosynthetic genes were identified in all conditions (Photosystem II D1), some were identified only on Earth (high-light-inducible genes) (Fig. 4b,c). To examine the significance of these variants, genome-wide Ka/Ks ratios were calculated. This ratio represents the fraction of non-synonymous variants (Ka) to synonymous variants (Ks) (Table 1). Overall, all conditions exhibited a Ka/Ks ratio < 1 indicating that variants were preferentially synonymous.

**Table 1:** Genome-wide Ka/Ks ratios.

Sample	Non-synonymous variants	Synonymous variants	Ka/Ks Ratio
Ground/UV	64	117	0.54
ISS/UV	57	131	0.43
ISS/Dark	44	113	0.38

*Ka* non-synonymous variant, *Ks* synonymous variant, *ISS* international space station, *UV* ultraviolet light, *Ground/UV* earth with UV exposure, *ISS/UV* ISS with UV exposure, *ISS/Dark* ISS without UV exposure.

When Ka/Ks ratios were calculated on a gene-by-gene basis with significant genes intersected across all experimental conditions, eight genes arose as regions of interest for examining the variant profiles in more depth. Four selected genes—a mobile element protein, *btuD*, *psbA*, and a transposase, displayed < 0.2 Ka/Ks ratios which indicates high density of synonymous variants (Table 2, Table S3). Three genes—two hypothetical proteins (proteins whose existence has been predicted but lack experimental evidence for in vivo expression) and a mobile element protein—displayed synonymous alterations only under certain conditions (Table 2, Table S3).

**Table 2:** Gene-by-gene Ka/Ks ratios.

Gene name	Feature ID in annotation	Ground/UV	ISS/UV	ISS/Dark
Hypothetical protein	fig|1177.46.peg.2854	1	1	**0.5**
Hypothetical protein	fig|1177.46.peg.2237	1	0	1
Mobile element protein	fig|1177.46.peg.2271	1	1	**0.17**
Mobile element protein	fig|1177.46.peg.1675	0	0	**0.05**
Photosystem II D1 (PsbA) protein	fig|1177.46.peg.5245	**0.17**	0	**0.15**
Transposase	fig|1177.46.peg.4085	**0.13**	**0.11**	**0.13**
Vitamin B12 ABC transporter, ATP-binding protein BtuD	fig|1177.46.peg.2110	**0.05**	0	0

*Ka* non-synonymous variant, *Ks* synonymous variant, *ISS* international space station, *UV* ultraviolet light, *Ground/UV* earth with UV exposure, *ISS/UV* ISS with UV exposure, *ISS/Dark* ISS without UV exposure.

Significant values are in bold.

We also examined mutations within non-coding regions of the genome. There was a minimal number of intragenic noncoding mutations—3 for Ground/UV, 4 for ISS/Dark, and 1 for ISS/UV—but Ground/UV and ISS/Dark shared 3 mutations in the exact same position with the same base pair substitution in genes coding for a hypothetical protein, LSU rRNA, and SSU rRNA. All 3 samples shared the mutation within the same gene encoding for the small subunit ribosomal rRNA, but the mutation in ISS/UV occurred in a different base pair (Supplemental File S1). All intragenic mutations occurred in the same ~ 35,000 bp region. There was a larger amount of intergenic noncoding mutations – 103 for Ground/UV, 159 for ISS/Dark, and 135 for ISS/UV. As with mutations seen in the coding regions of the genome, there appeared to be hotspots for intergenic noncoding mutations that were shared across the three conditions (Supplementary File S1). The distance to the closest gene was calculated for each intergenic mutation. The majority of intergenic mutations occurred within 10kbp of a gene—91/103 for Ground/UV, 113/159 for ISS/Dark, and 115/135 for ISS/UV. All mutations occurred within 80kbp across all conditions. As upstream regulatory elements are thought to be located up to 1 Mb from promoter regions of genes, it is possible that these mutations could have an impact on gene regulation, although it would require further work involving 3D conformation of the genome to confirm the full extent of this impact. When examining the specific base pair substitutions occurring in intergenic regions, a pattern begins to emerge (Table S6). A>G, G>A, C>T, and T>C are the most common reference to allele pairings across all experimental conditions for each respective reference nucleotide. A>G and T>C pairings would indicate increased GC content, and the reverse is true for C>T and G>A as it would decrease GC content. Furthermore, there are special cases of multiple base pair substitution in ISS/Dark and ISS/UV with 10 and 9 such instances respectively with Ground/UV having none, although no clear pattern emerges among these mutations.

**Figure 5:**
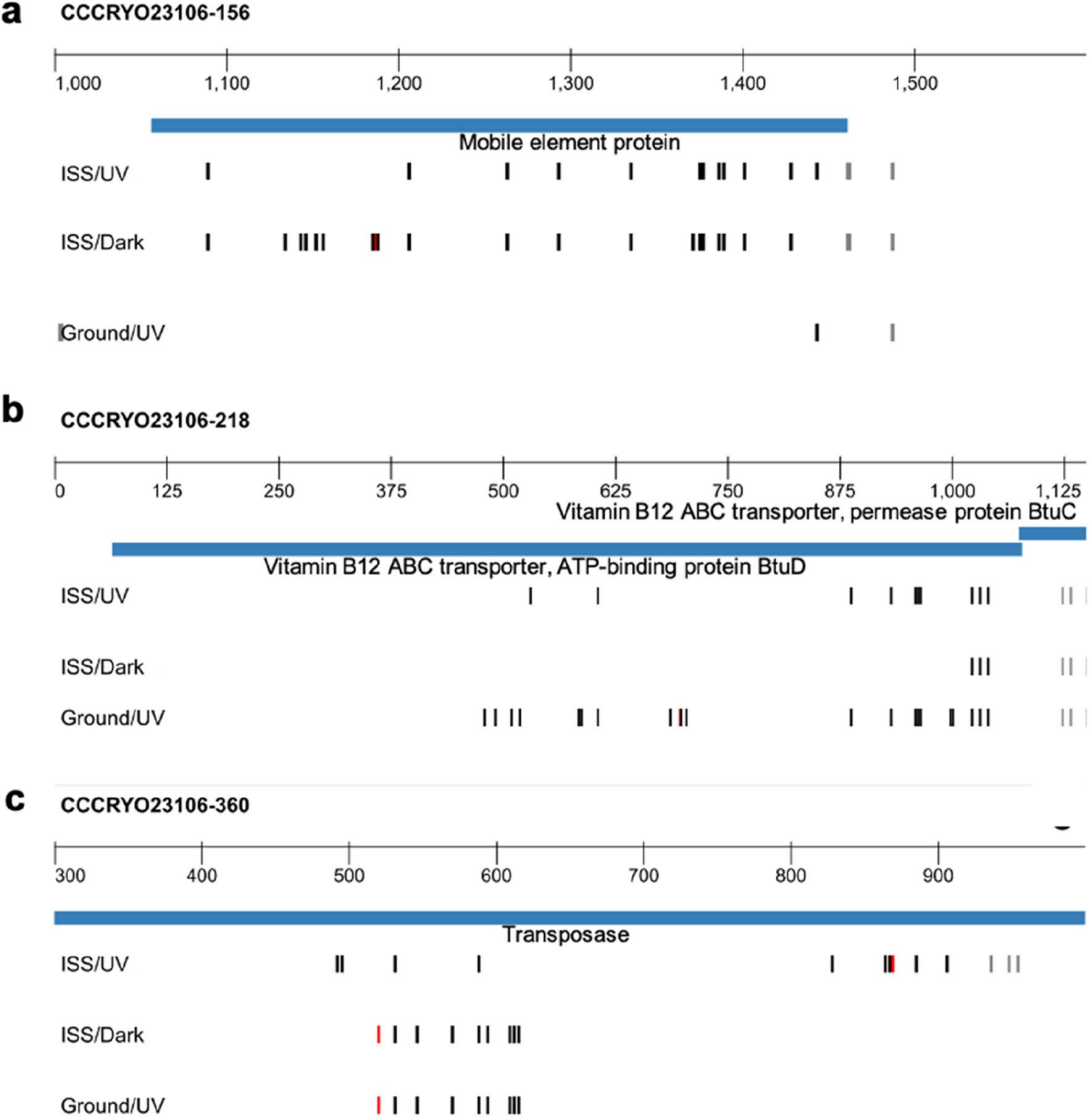
Selected synonymous (black) and non-synonymous (red) *Nostoc* sp. CCCryo 231-06 in BG11 medium variant profiles, where non-synonymous variants with respect to the consensus genome result in a change in amino acid. (**a**) Mobile element protein variant profile showing a high similarity between ISS/Dark and ISS/UV conditions. (**b**) BtuD variant profile showing similar variants for all three conditions, but more similarity between Ground/UV and ISS/UV throughout the whole sequence. (**c**) Transposase variant profile Ground/UV and ISS/Dark display the same sequence, while ISS/UV displays a different variant. *ISS* international space station, *UV* ultraviolet light, *ISS/UV* ISS with UV exposure, *ISS/Dark* ISS without UV exposure, *Ground/UV* earth with UV exposure.

These results suggest that these loci were preferentially altered. Variant profiles displayed alignment across experimental conditions, indicating the existence of variant hot spots (Figs. 4b,c, 5, Table S1). We calculated the probabilities of observing these variant patterns. A gene-by-gene analysis showed < 0.01% probability of the variants being random across conditions (Table S4). For genome wide variant space, the probability of the genes of interest randomly and independently emerging across all three conditions was < 10^–8^.

### Protein analysis

**Figure 6:**
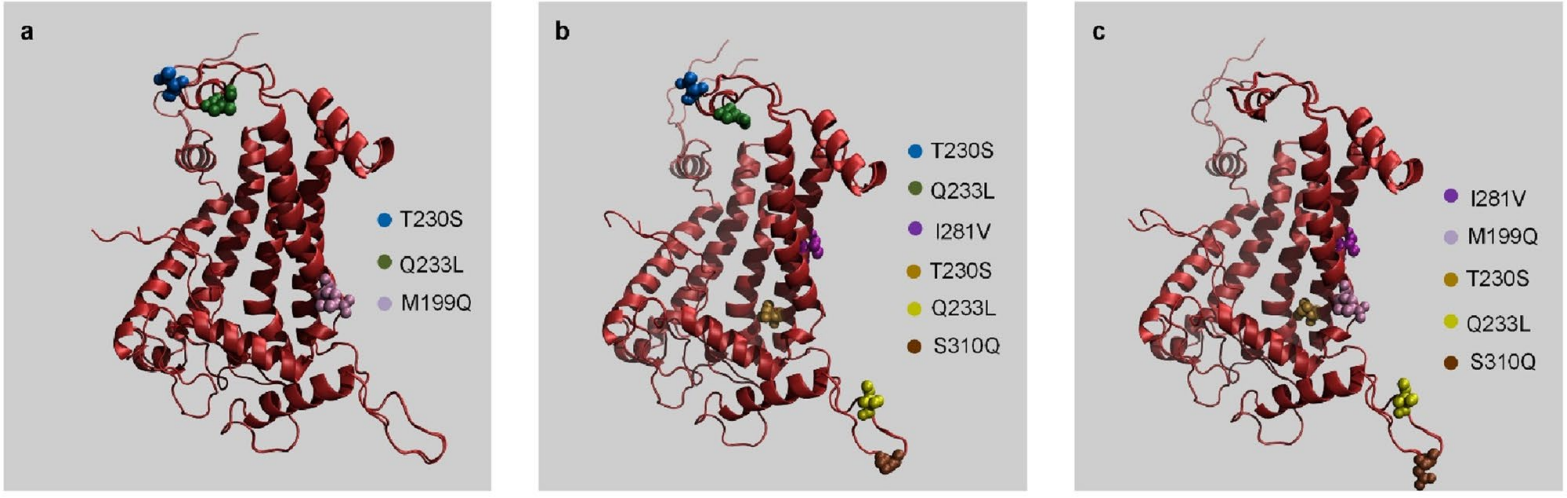
Non-synonymous sequence variants between the amino acid sequences from all three exposure conditions, overlaid on the respective predicted protein structures for the Photosystem II D1 (PsbA) protein. The marked locations represent the position of a non-synonymous variant between the sequences for the corresponding pair of conditions: (**a**) non-synonymous variants present in ISS/Dark but not ISS/UV conditions, (**b**) non-synonymous variants present in Ground/UV but not ISS/UV conditions, and (**c**) non-synonymous variants present in Ground/UV but not ISS/Dark conditions. *ISS* international space station, *UV* ultraviolet light, *ISS/Dark* ISS without UV exposure, *ISS/UV* ISS with UV exposure, *Ground/UV* earth with UV exposure.

Exposing *Nostoc* sp. CCCryo 231-06 to the tested environmental conditions resulted in synonymous and non-synonymous variants. While synonymous variants do not change the encoded protein and thus are thought to be silent, non-synonymous variants have a translational impact, which can be quantified on how they impact the encoded protein. Most of the variants detected represented synonymous changes. To determine whether the non-synonymous changes were likely to impact the protein structure of these factors, we performed a tertiary structure protein prediction analysis (RaptorX Structure Prediction). We focused on the photosystem II D1 (PsbA) protein encoding gene because it was the gene with the highest overall number of variants across the 3 experimental conditions (Table S3). PsbA exhibits fast, light-dependent turnover that is typically related to the repair of photo- inactivated PSII complexes. Previous studies suggest the differences in the coding region of the respective *psbA* gene are assigned to different intrinsic sensitivities of PSII complexes containing D1:1 or D1:2 to photo damage^28^. The predicted structures of the PsbA protein were compared between different experimental conditions and are presented in Fig. 6. For the samples on the ISS, non-synonymous variants of the protein occurred in samples kept in the dark, but not in those that experienced UV exposure (Figs. 4b, 6a). This suggests that the combinatorial nature of ionizing cosmic and UV radiations in ISS/UV may not have the same effect as each type of radiation alone. Compared with the samples exposed to UV radiation on the ISS, multiple variants emerged in the PsbA protein in the samples on the ground (Figs. 4b, 6b), which may indicate that the variants were due to unique aspects of Ground/UV, such as higher UV radiation levels (Table S1). For samples in all experimental conditions, no notable differences in protein structures were observed, except in the protein termini as marked in Fig. S1.

Sequence alignment of the PsbA protein is shown in Fig. S2. The alignment of the nucleotide sequences of the *psbA* gene (Fig. S3) across all conditions identified specific non-synonymous base pair substitutions shown in Fig. S1. Note that there were no insertions or deletions arising from different experimental conditions. Sequence alignment of a putative hemagglutinin-related protein is shown in Fig. S4. Sequence alignment of the nucleotide sequences of the putative hemagglutinin-related gene (Fig. S5) shows that only a few synonymous variants besides the non-synonymous variants highlighted in Fig. S4 emerged. Sequence alignment of a high light inducible protein (HLIP) family gene is shown in Fig. S6. In the sequence alignment of the nucleotide sequences of the HLIP family gene (Fig. S7), there was only a single non-synonymous change in the amino acid sequence. These results were found for all three experimental conditions.

## Discussion

The high purity of the returned samples that travelled through space on the ISS, i.e. the apparently reduced abundance of contaminating (non-*Nostoc*) bacteria in the original culture, indicates that the UV radiation in the near-Earth environment, exposure to galactic cosmic rays, trapped radiation belt particles, solar energy particles and secondary particle radiation^29^ likely exert complex effects that inflict lethal damage to a wide range of microorganisms. Compared to the simulated experimental conditions on Earth, radiation in space appeared to challenge the survival of microorganisms that are less resilient to these conditions. *Nostoc* species are capable of adapting to UV exposure to prevent radiation damage by producing UV-absorbing substances including shinorines, carotenoids or mycosporins^30^ in their natural habitats under increased irradiation. In addition, *Nostoc* sp. produces extracellular polysaccharides in response to stress conditions, enhancing its tolerance to desiccation and freezing temperatures^31^ in such extreme locations as rock surfaces, cold mountain and alpine soils or permafrost. These factors could play a significant role in maintaining the cellular structure and DNA integrity of the *Nostoc* sp. on BG11 which led to higher genome coverage.

Despite high preservation of the genome of *Nostoc* sp., we detected non-random variant spots and patterning in genes involved in biofilm production and photosynthetic activity, hemagglutinin and *psbA* in particular. Hemagglutinin-related genes mediate contact between cells, leading to colony formation and biofilm maturation^32^ protecting *Nostoc* colonies from desiccation. It is not clear if the identified associated loci have any effect on the colony formation and cell contact. However, colony-forming and growth experiments performed after the experiment in space (Fig. S8) showed no obvious phenotypic effects caused by the observed variants. We observed that colonies from ISS/UV conditions initially appeared bleached along the outer parts of the colonies, but only on BG11 medium or lunar regolith, which we attribute to excessive radiation and bleaching of the photosynthetic pigments with likely damage to the photosystem. Interestingly, this was not observed in colonies embedded within the martian regoliths (Mars-UV conditions). Colonies from those samples, no matter if from UV or dark conditions, looked intensely pigmented after rehydration and showed regrowth, young filaments and budding of new colonies, as well as heterocysts that are responsible for fixation of aerial nitrogen (Fig. S8). Differences in morphology, if at all, were noticeable between the substrates (BG11/Lunar versus P-MRS/S-MRS), but not between ground, dark or UV cultures from the ISS and thus, cannot be attributed to the variants detected. Differences in variants across substrates were unable to be examined accurately due to low genome coverage on Lunar and P-MRS/S-MRS analogs, which may have explained further these differences in morphology.

Photosystem-associated variants appeared in samples on BG11 both on the ISS and on Earth. The variants appeared in the *psbA* gene, which codes for the D1 polypeptide of the photosystem II (PS II) reaction center complex and is found in all photosynthetic organisms that perform oxygenic photosynthesis^33^. The PS II reaction center in general, is essential for the light reaction, fundamental for the conversion of light energy into chemical energy, the photolysis of water and the start of the electron chain reaction through the membranes of photosynthesizing organisms. This leads to the formation of valuable ATP necessary for the subsequent reactions in the Calvin cycle and fixation of CO_2_ derived carbon into carbohydrates (e.g. glucose, starch and other energy storage polysaccharides).

Based on our data, most of the genomic alterations observed were synonymous, and therefore no impact on survivability would be expected as a result. However, we cannot assess the survivability impact of the non-synonymous mutations, which primarily impacted protein termini structure. Follow up functional studies on these changes would have to be performed in order to make such an assessment. Space control samples that stayed on Earth were kept under Mars simulated conditions during the ground-based simulation, experiencing higher UV irradiation (up to 439 kJ/m^2^ of UV_200–400 nm_) than UV exposure at the ISS (up to 252 kJ/m^2^ of UV_200–400 nm_)^7,34^. During the ground-based simulation on Earth all three trays of the EXPOSE-R2 facility were exposed to an average UV fluence calculated for the wavelengths 200–400 nm by RedShift to represent the total irradiation fluences applied using a solar simulator (SOL2000, Dr. Hönle GmbH)^34^. However, individual compartments on the ISS facility experienced widely different UV fluences^7^ due to complex shading and positions of the ISS in relation to the sun, which could not be reproduced individually for the ground experiment. Phycobilisomes, the light-harvesting antennas in cyanobacteria, play a significant role in photo- and radiation protection^35^. Specifically, cyanobacteria are capable of uncoupling phycobilisomes from photosynthetic membranes which could be a first step in preventing radiation damage and overexcitation of the photosystems. Besides, stress conditions can also trigger photoprotection. These protective mechanisms prevent damage to the photosystems in cyanobacteria. However, we are not able to conclude if these variants would reduce or enhance the photosynthetic activity of the cell if fixed.

Comparing the final UV dose measured on the ISS for space and Mars samples with those on the ground (Table S1), the samples on Earth experienced both much stronger UV radiation intensity and final doses compared to the samples on the ISS. The samples on the ISS also received some shadowing from solar panels and other ISS modules while the ISS orbited the Earth. We note the extensive number of predicted changes in the *psbA* gene across all three exposures considered here, whereas neighboring genes were not as altered (with enough sequencing coverage for variant calling). This is possibly because the genes may have been preferentially damaged while in the desiccated state.

Due to the anhydrobiotic conditions during our experimental setting, we do not expect that significant biological activity, replication, or DNA repair activities took place. Therefore, our results are most likely solely a reflection of DNA damage occurred during the experimental conditions, as has been verified by other research groups under similar settings^36–38^. Single cell amplification and sequencing technologies have been recently deployed to characterize the “damagenome” profile in human cells^39^. This work represents a parallel technological effort to characterize genomic alterations in bacterial single cells. It is important to note the different external stresses across all experimental conditions. While ISS/UV and Ground/UV had different UV radiation exposure, ISS/UV and ISS/Dark shared exposure to cosmic radiation and microgravity. This provides some insight into the drivers of variant hotspots. ISS/UV and ISS/Dark were similar in the mobile element variant profile, while the *btuD* variant profile showed a stark similarity between Ground/UV and ISS/UV conditions. This gene is found in the vitamin B12 complex which has known sensitivity to UV exposure^40^, with reduced exposure under the ISS/Dark condition. Likewise, the *psbA* variant profile was exacerbated under Ground/UV conditions, which is also consistent with known sensitivity and degradation of the photosystem II, which includes inhibition of photosynthetic electron flow when under UV exposure^28^ and likely exposure to radiation damage. Exposure to UV irradiation has also been observed to drive differential growth and enzymatic activity in cyanobacteria, including other species of *Nostoc*^41^.

Interestingly, the transposase gene showed high similarity between variant profiles in ISS/Dark and Ground/UV, which shared no particular drivers for damage, although transposases are known to be reactive to both ionizing^42^ and UV radiation^43^. It is not clear why the variant profile between these 2 conditions shared such similarity, while ISS/UV conditions did not. It is likely that the combined effect of cosmic radiation and UV exposure in ISS/UV conditions generates a different outcome that is not simply combinatorial in nature. There are multiple explanations for observing consistent variants even under low probability. The flanking regions around the altered sites can increase the likelihood of point variations^44^, which might be synonymous or non-synonymous. Neighboring base pairs mechanistically influence the frequency and type of variants in different genomic landscapes. There also is a preferential codon usage, particular to the *Nostoc* species being examined^45^. The frequency of specific codon variant profiles as well as the DNA strand level mechanisms of the flanking motifs may contribute to the mechanisms for repeated variant events on a genomic level.

The combinatorial probabilities of genome-wide and gene-by-gene variants were used to evaluate the likelihood of observing certain variant profiles. The significance of these probabilities reduced the likelihood that the variant profiles observed across all three conditions were random. We therefore conclude that exposure of this *Nostoc* species to space conditions generates variants with preferential synonymous alterations of the biofilm and photosynthetic apparatus at the single cell level. Although all of us are used to contemplating a random mutation landscape upon which natural selection acts to select the fittest, our results suggest that the landscape may not always be random at the single cell level and under these unusual experimental conditions. Many variables may contribute to this outcome including a combination of DNA structure protected/vulnerable zones, bias in alterations caused by desiccation, UV and cosmic radiation for extended periods of time. Whatever those causes may be, the non-random nature of the alterations narrow the acting field for natural selection/genetic drift effects to subsequently occur when cells are revived. Our results suggest that under these particular experimental conditions, the non-random and largely synonymous alterations would create a scenario favoring stabilizing selection of the altered photosynthetic and biofilm genes upon revival.

## Conclusions

We have found that *Nostoc* CCCryo 231-06 displayed single cell variant profiles consistent with non-random genomic alterations on the ISS and simulated UV exposure on ground (Earth). The genetic variant hotspots were clustered in filamentous hemagglutinin and Photosystem II D1 loci. The variant patterns were repeatedly observed, diminishing the explanatory power of stochastic effects. Based on our analyses, the combined effect of complex cosmic radiation and UV exposure may result in synergistic damage effects, with a higher number of synonymous variants with simultaneous exposure to cosmic and UV radiations. The cause(s) and evolutionary implications of the non-random synonymous genomic substitutions observed at the single cell level under long-term cosmic exposure warrants further investigation, and may indicate that we have to rethink how evolution takes place at the single cell, and also population level.

## Supplementary Information

Supplementary Information.

## Data Availability

Genome data for *Nostoc* sp. CCCryo 231-06 has been deposited at NCBI (BioProject accession number PRJNA721463). The Whole Genome Shotgun project has been deposited at DDBJ/ENA/GenBank under the accession JAHCSU010000000. All data needed to evaluate the conclusions in the paper are present in the paper and/or the Supplementary Materials S1.
