# Supplementary material for: Non-random genetic alterations in the cyanobacterium Nostoc sp. exposed to space conditions
Source: Sci Rep. 2022 Jul 22;12:12580. doi: 10.1038/s41598-022-16789-w (PMC9307615; doi:10.1038/s41598-022-16789-w)
Supplement: Supplementary file 1 — Supplementary Information. [file 41598_2022_16789_MOESM1_ESM.docx]

**Supplemental Information**

**Non-random genetic alterations in the cyanobacterium Nostoc sp. exposed to space conditions**

Yuguang Liu^#1,2,13^, Patricio Jeraldo^#1,2^, William Herbert^2,14^, Samantha McDonough^3^, Bruce Eckloff^3^, Jean-Pierre de Vera^4^, Charles Cockell^5^, Thomas Leya^6^, Mickael Baqué^7^, Jin Jen^3^, Dirk Schulze-Makuch^*8-11^, Marina Walther-Antonio^*^ ^1,2,12^

^1^Department of Surgery, Division of Surgical Research, Mayo Clinic, Rochester, MN 55905, USA

^2^Microbiome Program, Center for Individulized Medicine, Mayo Clinic, Rochester, MN 55905, USA

^3^Medical Genome Facility, Center for Individualized Medicine, Mayo Clinic, Rochester, MN, USA

^4^Space Operations and Astronaut Training, Microgravity User Support Center (MUSC), German Aerospace Center (DLR), Linder Höhe, 51147 Köln, Germany

^5^School of Physics and Astronomy, University of Edinburgh, Edinburgh EH9 3FD, UK

^6^Fraunhofer Institute for Cell Therapy and Immunology, Branch Bioanalytics and Bioprocesses IZI-BB, 14476, Potsdam, Germany

^7^Astrobiological Laboratories, Planetary Laboratories Department, Institute of Planetary Research, German Aerospace Center (DLR), 12489 Berlin, Germany^8^Astrobiology Group, Center of Astronomy and Astrophysics, Technische Universität Berlin, 10623 Berlin, Germany

^9^GFZ German Research Center for Geosciences, Section Geomicrobiology, Potsdam, Germany.

^10^Leibniz-Institute of Freshwater Ecology and Inland Fisheries (IGB), Department of Experimental Limnology, Stechlin, Germany.

^11^School of the Environment, Washington State University, Pullman, Washington, USA.

^12^Department of Obstetrics and Gynecology, Mayo Clinic, Rochester, MN 55905, USA

^13^Department of Immunolgy, Mayo Clinic, Rochester, MN 55905, USA

^14^Mayo Clinic Graduate School of Biomedical Sciences, Mayo Clinic, Rochester, MN 55905, USA

**Strain origin and pre-flight sample preparation**

The antarctic strain CCCryo 231-06 (= UTEX EE21; CCMEE 391) of the cyanobacterium *Nostoc* sp*.* (hereafter, for readability addressed as "the *Nostoc*" only) was obtained from the Culture Collection of Cryophilic Algae (CCCryo) at the Branch Bioanalytics and Bioprocesses of the Fraunhofer Institute for Cell Therapy and Immunology IZI-BB in Potsdam, Germany. It was originally collected by E.I. Friedmann in 1979 at Gneiss Point in Victoria Land, Antarctica. Since 2006 it is cultured and maintained at the CCCryo biobank. Samples derived from this living strain were used in BIOMEX experiments on the ISS (= space) and in ground simulation chambers. Details of BIOMEX experimental parameters are provided below. The strain was not axenic, i.e. besides the *Nostoc* cyanobacterium itself it contained other bacteria and possibly fungi, yet no other cyanobacterial species.

For the BIOMEX (see below) experiments strain CCCryo 231-06 was first grown in liquid BG11 medium, a typical culture medium widely used for cyanobacteria, at 12 °C to obtain sufficient biomass. For the preparation of the samples used for the BIOMEX experiments desiccated substrate pellets carrying the desiccated *Nostoc* colonies were needed. For this purpose, agar plates made from the BG11 medium, lunar regolith analog, phyllosilicatic martian regolith (P-MRS) and sulfatic martian regolith (S-MRS) were prepared. For the regolith agar plates, each of the regoliths was added at 12.5% to aqua bidest., heated in a microwave and then boiled for 1 h at 100 °C on a hot plate, cooled to room temperature and then the pH of the suspension was adjusted to a value of 8.5 (the pH of the BG11 medium used) with sodium hydroxide solution. Agar agar (Serva Electrophoresis GmbH, Heidelberg, Germany) was added at 2 % (w/v) to each preparation, the suspension was again heated in a microwave and then autoclaved for 20 min. at 121°C. All three regolith agar suspensions as well as the agarized BG11 medium were poured to plates (62 mm polystyrene Petri dishes) and stored for a minimum of 5 days prior to use. Petri dishes with BG11 agar were prepared as usual (see medium recipe on the strain's CCCryo website: <http://cccryo.fraunhofer.de/web/strains/search/231>).

*Nostoc* sp. CCCryo 231-06 from the liquid cultures was inoculated onto the respective agar plates and cultured at 12 °C irradiated with fluorescent tubes (type cool white and fluora) at photon flux rates of 20-50 µE m^-2^ s^-1^ in a light cycle of 16:8 hours (light:dark). When the *Nostoc* colonies had grown to a sufficient number they were desiccated in the course of 9 days in a desiccator. To prevent curling of the rims of the agar, autoclaved sample grids self-made from POM (polyoxymethylene) fitting into the Petri dishes were placed onto the agar accommodating the *Nostoc*. The grids contained several 13 mm holes to facilitate drying, but prevent curling or folding of the drying agar. The desiccation process over silica gel lasted 9 days and started at a low pressure of 30,000 Pa for 3 days, followed by 3 days at 20,000 Pa and was finalized after 3 days at 10,000 Pa. When this process was complete and the agar with the *Nostoc* was dry, the POM grid was removed and pellets of 12 mm in diameter were punched out with a sterilized cork borer. The dry sample pellets were sent to the DLR (Deutsches Zentrum für Luft- und Raumfahrt, German Aerospace Center) in Cologne in sterile multiwell dishes, and there they were accommodated under sterile conditions in the sample holders to be fixed at the designated positions in the carrier tray, which later was to be exposed on the outside of the Zvezda module on the ISS during the BIOMEX experiment, or kept in the Martian simulation chamber at DLR with/without UV exposure for (1). After return of all samples to Fraunhofer IZI-BB parts were sent to the authors from Mayo Clinic where they were rehydrated briefly and intact single-cells from each sample were selected for single cell genomic analysis.

**BIOMEX experimental parameters**

BIOMEX is a European Space Agency (ESA) space exposure experiment in the EXPOSE-R2 facility outside the Zvezda module on the ISS. The cultured microorganism and regolith analogs and terrestrial mineral mixtures were exposed to space and simulated Mars-like conditions in the EXPOSE-R2 facility(1). Two trays were used in the BIOMEX experiment on the EXPOSE-R2 facility to provide two distinct exposure conditions regarding the UV regimes and gas composition: tray 1 for space conditions and tray 2 for Mars-like conditions. BG11 and Lunar pellets were exposed in the tray 1 to space conditions, and S-MRS and P-MRS pellets in the tray 2 to Mars-like conditions. Specifically, the space condition tray was evacuated once installed outside the ISS at a vacuum pressure of 4.1 × 10^-5^ Pa. Samples under space conditions were covered with MgF_2_ windows transmitting solar extraterrestrial electromagnetic radiation of λ>110 nm. For the simulation of Mars-like conditions, a Mars-like atmosphere composed of 95.55% CO_2_, 2.7% N_2_, 1.6% Ar, 0.15% O_2_, and ∼370 ppm H_2_O at 980 Pa was used, and the samples were covered by quartz windows to adjust the LEO extraterrestrial UV spectrum to a Mars-like one transmitting wavelengths of λ>200 nm. Other parameters of the martian environment (such as gravity, temperature regime, and ionizing radiations) cannot be simulated in LEO. For all the trays in the EXPOSE-R2 facility the temperature varied depending on the exposition of the sample trays to the Sun but was kept above -25°C with heater elements to protect the electronics. The recorded temperatures in tray 1 (space tray) cycled between -20°C at minimum and +47.2°C at maximum while in tray 2 (Mars tray) they cycled between -19.7 ºC at minimum(2) and +47.1°C at maximum. Furthermore, the whole EXPOSE-R2 facility experienced the microgravity environment at the ISS and the ionizing radiation environment found in LEO at the altitude of the ISS: protons from the inner radiation belt, galactic cosmic rays and outer radiation belt electrons for a total mission dose of ~500 mGy(2). Finally, the samples were accommodated in stacks of two layers in all trays to allow an identical exposure of all the samples in one compartment to the same conditions (space or Mars-simulated) except solar UV radiations: the top layer is thus denoted “UV” and the bottom “Dark”.

The experimental variables included UV radiation attenuated by 0.1% transmission filters (composed of MgF_2_ for space conditions and quartz filters for Mars conditions), additional dark conditions, as well as the four aforementioned media (BG11 medium, lunar regolith analog, P-MRS and S-MRS). As a comparative study, the same samples were also exposed in identical hardware (with top and bottom layers) in experimental chambers on the ground, in the Planetary and Space Simulation Facilities (PSI) at DLR Cologne, simulating the space environment outside the ISS and Mars-like conditions (except for UV<200nm, microgravity and ionizing radiation) during the Mission ground reference (MGR) and are thus called “Ground”. The BIOMEX samples were on the ISS for 23 months, placed outside the ISS for 17 months, and exposed to UV for 15 months (10/22/2014 – 2/3/2016). A brief summary of exposure conditions for each condition is provided in Table S1 and further details can be found in previous work(3). Details of the components of regolith analogs were reported in previous work(3) and are briefly summarized in Table S2. These samples were returned to Earth and collected for post-flight processing (see supplemental information) before SC-WGA in a microfluidic device for sequencing and analysis. The overall workflow is illustrated in Figure 1.

**BIOMEX samples post-flight processing**

The samples were placed in the carrier tray and mounted on the outside of the ISS, exposed to UV for 15 months, and returned to Earth in Kazakhstan in a Soyuz capsule. Before return from the ISS the gas valves of tray 1 (space tray) were closed so that the gas conditions remained the same as during the flight. Tray 2 (Mars tray) retained the Mars simulated atmosphere for the whole duration of the mission until de-integration. The samples were subjected to 25°C outside temperature at landing, and were first transported to Moscow, Russia and then to Munich, Germany at ambient temperatures. After customs in Germany, they were stored in a 15°C temperature-controlled container and transported to the DLR in Cologne. At DLR, the trays were placed in an anaerobic workbench with nitrogen gas and was equilibrated to ambient pressure. The trays were dissembled and the individual samples from the top (ISS/UV) and bottom layer (ISS/Dark) were de-integrated from the carriers into their original multiwells, in which they were forwarded to DLR when the mission was prepared 24 months earlier. The samples, each on their respective medium pellet, were returned to Fraunhofer IZI-BB still in desiccated state. They were then handled under sterile conditions in a clean bench to minimize contamination. The glue used to fix the pellets on the sample holders was removed mechanically with alcohol sterilized forceps. Each pellet with adhering *Nostoc* colonies was then cut into quarters with an alcohol sterilized pair of scissors. All samples were stored in a desiccator at 800 mbar until further post-flight experiments. One quarter of each sample was dispatched to the authors from the Mayo Clinic for SC-WGA and subsequent sequencing. Samples from the ground simulation were treated accordingly when de-integrated from their sample holders. For the reference samples living (non-desiccated) biomass from the CCCryo biobank was used.

**Sample rehydration for SC-WGA in microfluidic device**

At the Mayo Clinic the desiccated samples (one quarter of each original sample) were rehydrated in 200 μL sample diluent (0.08% Pluronic F127 (Sigma Aldrich) in Phosphate Buffer Saline (PBS)) in 1.5 mL Eppendorf tubes followed by mild micro-pestling for 30 s to resuspend the cells. The final concentration reached ~10 cells μL^-1^ to facilitate single-cell trapping in the microfluidic device. 2 μL of the rehydrated cells were used to check the cell concentration under the microscope. Note that remaining debris from agarized medium or pellets from the regoliths used in the experiments were observed, but disregarded.

**Nostoc lysis and WGA**

For this experiment we used Qiagen REPLI-g Single Cell Kit with optimized lysis conditions. Three cycles of heat-shock were performed by alternately placing the microfluidic chip on a 65˚C hotplate and a -20˚C cold block for 2 min each. A custom-made lysis buffer containing 0.5 mM of EDTA, 200 U µL^-1^ of Ready-Lyse lysozyme (Epicenter), 200 mM Dithiothreitol (DTT) (BioRad) was introduced into the microchambers and the microfluidic device was incubated on a hotplate at 37˚C for 2 hrs. D2 lysis and DNA denature buffer supplied in the Single Cell Kit was introduced and incubated on a hotplate at 37˚C for 1 hr, followed by the addition of the neutralizing buffer to terminate DNA denaturation at room temperature. The polymerase added to the reaction chambers, and the device were placed on a hotplate at 32˚C for 16 hrs. The amplification was terminated by incubating the microfluidic device at 65˚C for 3 min and cooled on ice. The amplified DNA was collected from the outlet ports of the device through gel-loading pipet tips and transferred into 96 microwell plates for downstream processing. All the supplies and reagents were filtered (0.2 μm), autoclaved or UV-sterilized, except for the DNA polymerase.

**Library construction and sequencing**

DNA was normalized to 10 ng in 50 µl of low-TE buffer and sheared in a microTUBE AFA fiber plate using a Covaris LE220 instrument (Covaris, Woburn, MA, USA) to acquire fragments of approximately 200 bp. After shearing, libraries were prepared using NEBNext Ultra II DNA Library Prep Kit for Illumina (New England Biolabs, Ipswich, MA, USA) and dual-indexed using NEBNext Multiplex Oligos (New England Biolabs) according to manufacturer’s instructions. SPRIselect Beads (Beckman Coulter, Indianapolis, IN, USA) were used for all library clean-up steps. Libraries were sequenced generating 150 bp paired end reads using an Illumina HiSeq 4000 (Illumina, San Diego, CA, USA).

**Preprocessing of sequenced reads**

We converted the bam files to fastq files using SAMtools version 1.9(4). To enhance the quality and reliability of sequencing reads for downstream analysis, we trimmed sequencing adapters and trimmed low-quality bases using Atropos version 1.1.19(5). We also removed putative contaminants including environmental bacteria, fungi and human contaminants using BioBloomTools version 2.1.1(6).

**Metagenomics analysis**

To study the composition of the samples to better gauge the success of the amplification step, we used Kraken2 version 2.0.8-beta, together with Bracken v2.5 to obtain the taxonomic profile of the samples, using the pre-processed reads from all samples(7). We used a database of NCBI RefSeq genomes from Bacteria, Archaea, Fungi, Viruses and a human genome reference, as present in January 2019.

**Base quality score recalibration**

As the samples were sequenced in an Illumina HiSeq 4000 instrument, the returned reads had the PHRED scores for the base qualities “binned” into discrete groups, instead of a continuum of values from 0 to 42, and this can pose a problem when calling variants(8), therefore requiring a procedure for base quality score recalibration. First, we created a single metagenomic assembly per sequencing lane using MEGAHIT. Then, using the BBMap from the BBTools suite, we mapped the reads onto this assembly, creating a SAM file. Then, for each fastq file for that sequencing lane, we use the BBDuk tool to recalibrate them using the SAM file for recalibration information, thus obtaining fastq files with recalibrated PHRED scores.

**Visualization of sequence alignments**

The nucleotide and amino acid sequences of proteins of interest were visualized using ESPript 3(9). No multiple sequence alignment software was necessary as the sequences were of the same length, with no insertions or deletions present. In the case of the amino acid sequences of the photosystem II D1 protein PsbA, the alignment was decorated with its predicted secondary structure, as obtained for the consensus reference sequence.

**Table S1.** List of *Nostoc* CCCryo 231-06 samples exposed to different conditions and the number of single cells sequenced. LD = hours light:dark; ISS – International Space Station; PAR – Photosynthetic Active Radiation; S-MRS – Sulfatic Martian Regolith; P-MRS – Phyllosilicatic Martian Regolith; UV – Ultraviolet Light.

| Sample No. | Sample name | Exposure condition | # of single cells |
| --- | --- | --- | --- |
| 1 | 231-06 Control = reference | Original live strain from CCCryo biobank, 3 °C, no UV, PAR from Valoya LED lights type L18 spectrum NS12 at 4-10 µE m^-2^ s^-1^, light cycle 16:8 LD | 4 |
| Samples on the ISS | | | |
| 2 | 1-1-t-05: 231-06, BG11 | space, UV_200-400nm_: 171 kJ/m^2^, UV+PAR: 2960 kJ/m², MgF_2_ filtered | 4 |
| 3 | 1-1-t-06: 231-06, Lunar | space, UV_200-400nm_: 207 kJ/m^2^, UV+PAR: 3550 kJ/m², MgF_2_ filtered | 4 |
| 4 | 1-1-b-05: 231-06, BG11 | space, dark | 4 |
| 5 | 1-1-b-06: 231-06, Lunar |  | 4 |
| 6 | 2-1-t-05: 231-06, S-MRS | Mars, UV_200-400nm_: 208 kJ/m^2^, UV+PAR: 2650 kJ/m², quartz filtered | 4 |
| 7 | 2-1-t-06: 231-06, P-MRS | Mars, UV_200-400nm_: 252 kJ/m^2^, UV+PAR: 3180 kJ/m², quartz filtered | 3 |
| 8 | 2-1-b-05: 231-06, S-MRS | Mars, dark | 4 |
| 9 | 2-1-b-06: 231-06, P-MRS |  | 4 |
| Samples on the ground | | | |
| 10 | 1-1-t-05: 231-06, BG11 | ground (with space simulation), UV_200-400nm_ (439 kJ/m^2^), MgF_2_ filtered | 4 |
| 11 | 1-1-t-06: 231-06, Lunar |  | 4 |
| 12 | 2-1-t-05: 231-06, S-MRS | ground (with Mars simulation), UV_200-400nm_ (437 kJ/m^2^), quartz filtered | 4 |
| 13 | 2-1-t-06: 231-06, P-MRS |  | 1 |

**Table S2.** A list of analog mineral mixtures. P-MRS – Phyllosilicatic Martian Regolith; S-MRS – Sulfatic Martian Regolith.

| Component | Lunar (*wt%*) | P-MRS (*wt%*) | S-MRS (*wt%*) |
| --- | --- | --- | --- |
| Gabbro (Groß-Bieberau, Germany) |  | 3 | 32 |
| Dunite—Olivine Fo_96_ (Åheim, Norway) | 5.7 | 2 | 15 |
| CPx—Diopside (Kragerö, Norway) | 8.9 |  |  |
| OPx—Hyperstehn (Egersund, Norway) | 5.7 |  |  |
| Anorthosite—Plagioclase (Larvik, Norway) | 66.8 |  |  |
| Quarzite (Bayerischen Wald, Germany) |  | 10 | 3 |
| Apatite (Minas Gerais, Brasil) | 1.1 |  |  |
| Hematite (Cerro Bolivar, Venezuela) |  | 5 | 13 |
| Illmenite (Flekkefiord, Norway) | 1.1 |  |  |
| Iron (Fe) | 1.3 |  |  |
| Montmorillonite (Hallertau, Germany) |  | 45 |  |
| Chamosite (Nucic, Czech Republic) |  | 20 |  |
| Kaolinite (Hirschau, Germany) |  | 5 |  |
| Siderite (Hüttenberg, Austria) |  | 5 |  |
| Hydromagnesite (Albaner Berge, Italy) |  | 5 |  |
| Goethite (Salchendorf, Germany) |  |  | 7 |
| Gypsum (Nüttermoor, Germany) |  |  | 30 |
| Volcanic slag (Aeolian islands, Italy) | 9.4 |  |  |

**Table S3** Gene-by-gene non-synonymous and synonymous variant counts. ISS – International Space Station; UV – Ultraviolet Light.

| Condition | | Ground/UV | | ISS/UV | | ISS/Dark | |
| --- | --- | --- | --- | --- | --- | --- | --- |
| Gene name | feature_id in genome | Non-synonymous | Synonymous | Non-synonymous | Synonymous | Non-synonymous | Synonymous |
| Mobile element protein | fig\|1177.46.peg.1675 | 0 | 1 | 0 | 12 | 1 | 19 |
| Vitamin B12 ABC transporter ATP-binding protein BtuD | fig\|1177.46.peg.2110 | 1 | 20 | 0 | 10 | 0 | 3 |
| hypothetical protein | fig\|1177.46.peg.2237 | 5 | 5 | 1 | 1 | 1 | 2 |
| Mobile element protein | fig\|1177.46.peg.2271 | 1 | 1 | 1 | 1 | 1 | 6 |
| hypothetical protein | fig\|1177.46.peg.2854 | 2 | 2 | 0 | 2 | 2 | 2 |
| Transposase | fig\|1177.46.peg.4085 | 1 | 8 | 1 | 9 | 1 | 8 |
| Photosystem II D1 (PsbA) protein | fig\|1177.46.peg.5245 | 6 | 29 | 0 | 13 | 3 | 13 |

**Table S4** Combinatoric probabilities for selected genes. ISS – International Space Station; UV- Ultraviolet Light.

| Genes | Gene name | Length (Variant positions) | Comparing | Shared Variants | Combinatoric probability |
| --- | --- | --- | --- | --- | --- |
| CCCRYO23106-156:1057-1461 | Mobile element protein | 404 | ISS/Dark-ISS/UV | 11 | 9.79E-22 |
|  |  |  | ISS/Dark-Ground/UV | 0 | 1 |
|  |  |  | Ground/UV-ISS/UV | 1 | 2.48E-3 |
|  |  |  | All 3 | 0 | 1 |
| CCCRYO23106-218:65-1078 | BtuD | 1013 | ISS/Dark-ISS/UV | 3 | 5.79E-09 |
|  |  |  | ISS/Dark-Ground/UV | 3 | 5.79E-09 |
|  |  |  | Ground/UV-ISS/UV | 9 | 3.35E-22 |
|  |  |  | All 3 | 3 | 5.79E-09 |
| CCCRYO23106-231:6-1349 | Hypothetical protein | 1343 | ISS/Dark-ISS/UV | 0 | 1 |
|  |  |  | ISS/Dark-Ground/UV | 3 | 2.48E-09 |
|  |  |  | Ground/UV-ISS/UV | 0 | 1 |
|  |  |  | All 3 | 0 | 1 |
| CCCRYO23106-233:26131-26295 | Mobile element protein | 164 | ISS/Dark-ISS/UV | 2 | 7.48E-05 |
|  |  |  | ISS/Dark-Ground/UV | 2 | 7.48E-05 |
|  |  |  | Ground/UV-ISS/UV | 2 | 7.48E-05 |
|  |  |  | All 3 | 2 | 7.48E-05 |
| CCCRYO23106-271:22818-23291 | Hypothetical protein | 473 | ISS/Dark-ISS/UV | 2 | 8.96E-06 |
|  |  |  | ISS/Dark-Ground/UV | 3 | 5.71E-08 |
|  |  |  | Ground/UV-ISS/UV | 2 | 8.96E-06 |
|  |  |  | All 3 | 2 | 8.96E-06 |
| CCCRYO23106-360:82-1083 | Transposase | 1001 | ISS/Dark-ISS/UV | 2 | 2.00E-06 |
|  |  |  | ISS/Dark-Ground/UV | 9 | 3.73E-22 |
|  |  |  | Ground/UV-ISS/UV | 2 | 2.00E-06 |
|  |  |  | All 3 | 2 | 2.00E-06 |
| CCCRYO23106-370:3-422 | Transferase | 419 | ISS/Dark-ISS/UV | 1 | 2.39E-04 |
|  |  |  | ISS/Dark-Ground/UV | 1 | 2.39E-04 |
|  |  |  | Ground/UV-ISS/UV | 1 | 2.39E-04 |
|  |  |  | All 3 | 1 | 2.39E-04 |
| CCCRYO23106-461:16363-17445 | PsbA | 1082 | ISS/Dark-ISS/UV | 0 | 1 |
|  |  |  | ISS/Dark-Ground/UV | 11 | 1.77E-26 |
|  |  |  | Ground/UV-ISS/UV | 9 | 1.85E-22 |
|  |  |  | All 3 | 0 | 1 |

**Table S5** Location and genes of deleterious variants. ISS – International Space Station; UV – Ultraviolet Light.

| Sample | Contig | Gene | Position |
| --- | --- | --- | --- |
| ISS/Dark | CCCRYO23106-59 | Hypothetical protein | 381 |
|  |  |  | 414 |
|  |  |  | 552 |
|  | CCCRYO23106-99 | Intergenic region | 1695 |
|  | CCCRYO23106-234 | Mobile element protein | 3353 |
|  |  |  | 4055 |
|  | CCCRYO23106-271 | Intergenic region | 16368 |
|  | CCCRYO23106-302 | Intergenic region | 75 |
|  | CCCRYO23106-412 | Intergenic region | 514 |
|  | CCCRYO23106-428 | Putative hemagglutinin-related protein | 43802 |
|  |  |  | 44672 |
|  |  |  | 44706 |
|  | CCCRYO23106-461 | PsbA | 16957 |
|  |  |  | 17015 |
|  | CCCRYO23106-682 | Intergenic region | 21591 |
| ISS/UV | CCCRYO23106-332 | Intergenic region | 522 |
|  | CCCRYO23106-412 | Intergenic region | 514 |
|  | CCCRYO23106-609 | Intergenic region | 1216 |
|  | CCCRYO23106-614 | Intergenic region | 370 |
| Ground/UV | CCCRYO23106-234 | Mobile element protein | 3353 |
|  | CCCRYO23106-461 | PsbA | 17015 |
|  |  |  | 17287 |

**Table S6** Specific base pair substitutions of point mutations in intergenic regions

|  | A | | | G | | | C | | | T | | |
| --- | --- | --- | --- | --- | --- | --- | --- | --- | --- | --- | --- | --- |
|  | Ground/UV | ISS/Dark | ISS/UV | Ground/UV | ISS/Dark | ISS/UV | Ground/UV | ISS/Dark | ISS/UV | Ground/UV | ISS/Dark | ISS/UV |
| A |  |  |  | 30 | 28 | 21 | 3 | 8 | 6 | 4 | 6 | 10 |
| G | 15 | 16 | 15 |  |  |  | 5 | 12 | 8 | 3 | 4 | 4 |
| C | 4 | 10 | 8 | 3 | 4 | 6 |  |  |  | 16 | 23 | 20 |
| T | 5 | 8 | 5 | 3 | 13 | 7 | 12 | 14 | 16 |  |  |  |


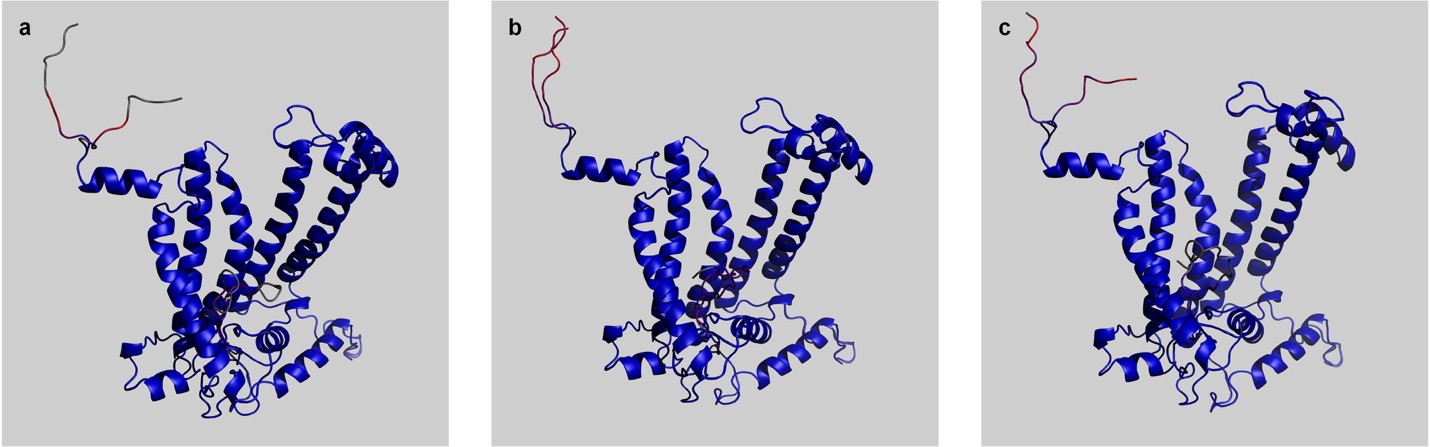


**Figure S1.** Differences in the predicted structures for the photosystem II D1 (PsbA) protein among all three exposure conditions. Color represents root-mean-square deviation of atomic positions, from zero differences (in blue) to largest difference (in red). Comparisons are for (a) ISS/Dark and ISS/UV conditions, (b) Ground/UV and ISS/UV conditions, and (c) Ground/UV and ISS/Dark conditions. No notable structural differences are observed except in the protein termini. ISS – International Space Station; UV- Ultraviolet Light.


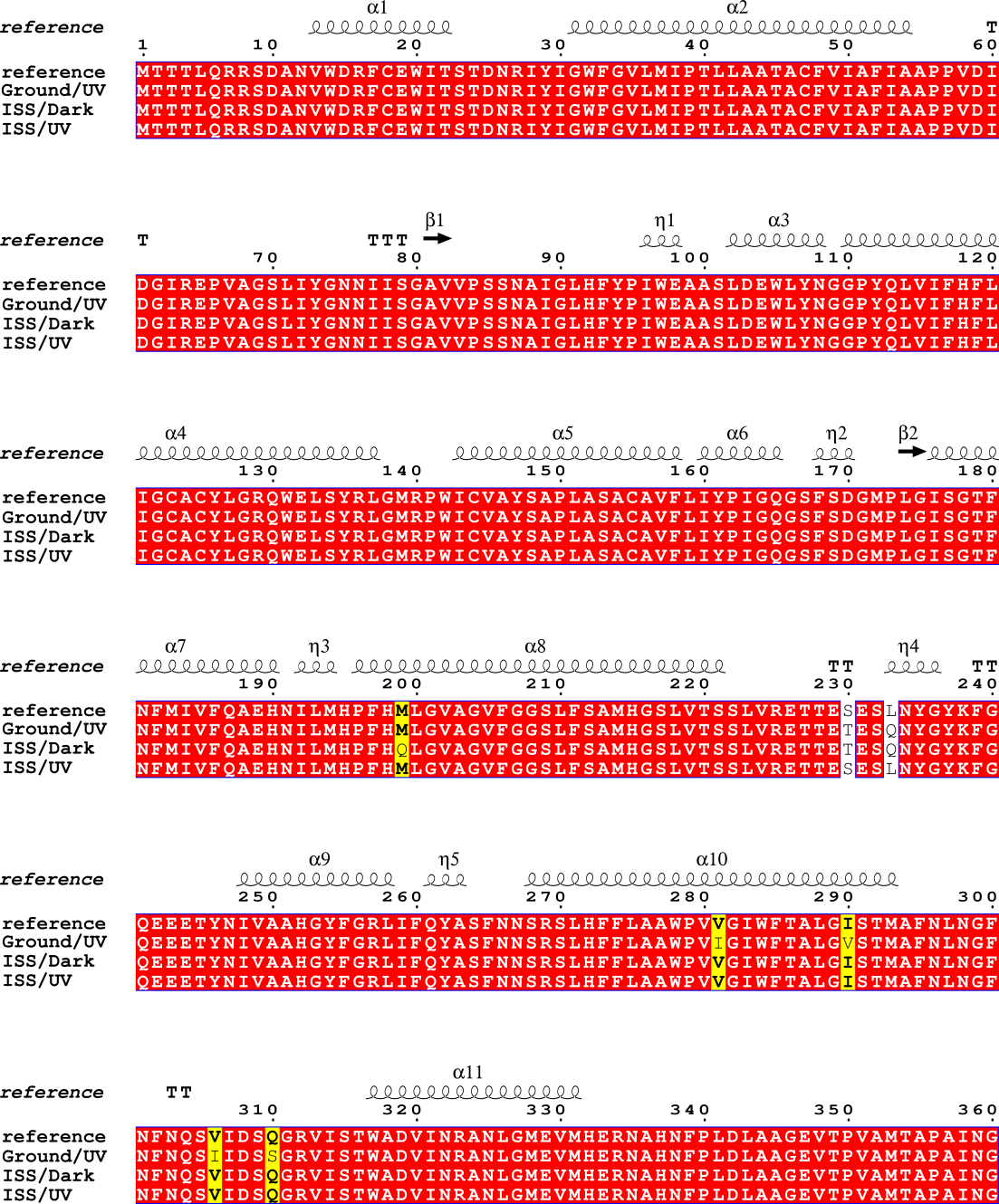


**Figure S2.** Multiple sequence alignment of the photosystem II D1 (PsbA) protein (CCCRYO23106-461:16363-17445) for all three experimental conditions, and the consensus amino acid sequence as the non-treated reference. The alignment is also annotated with the predicted secondary structure of the consensus sequence, showing α-helices (α), β-sheets (β), 3_10_-helices (η), strict β-turns (TT) and strict α-turns (TTT). Yellow columns represent variants unique to a single experimental condition while white columns represent variants shared across multiple experimental conditions. ISS – International Space Station; UV – Ultraviolet Light.


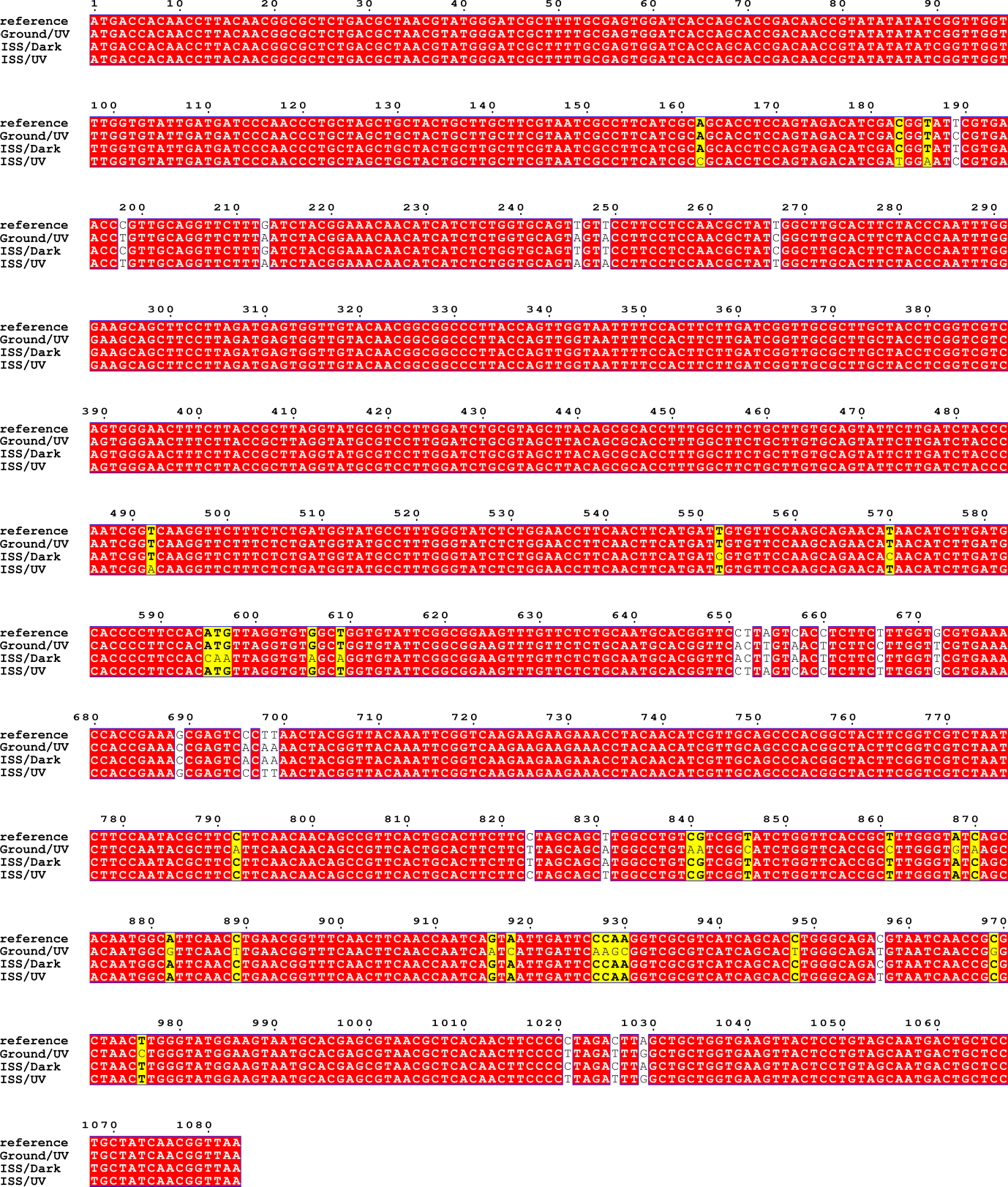


**Figure S3.** Multiple sequence alignment of the nucleotide sequences of the photosystem II D1 (*psbA*) gene (CCCRYO23106-461:16363-17445) for all three experimental conditions and the consensus sequence. Yellow columns represent variants unique to a single experimental condition while white columns represent variants shared across multiple experimental conditions. ISS – International Space Station; UV – Ultraviolet Light.


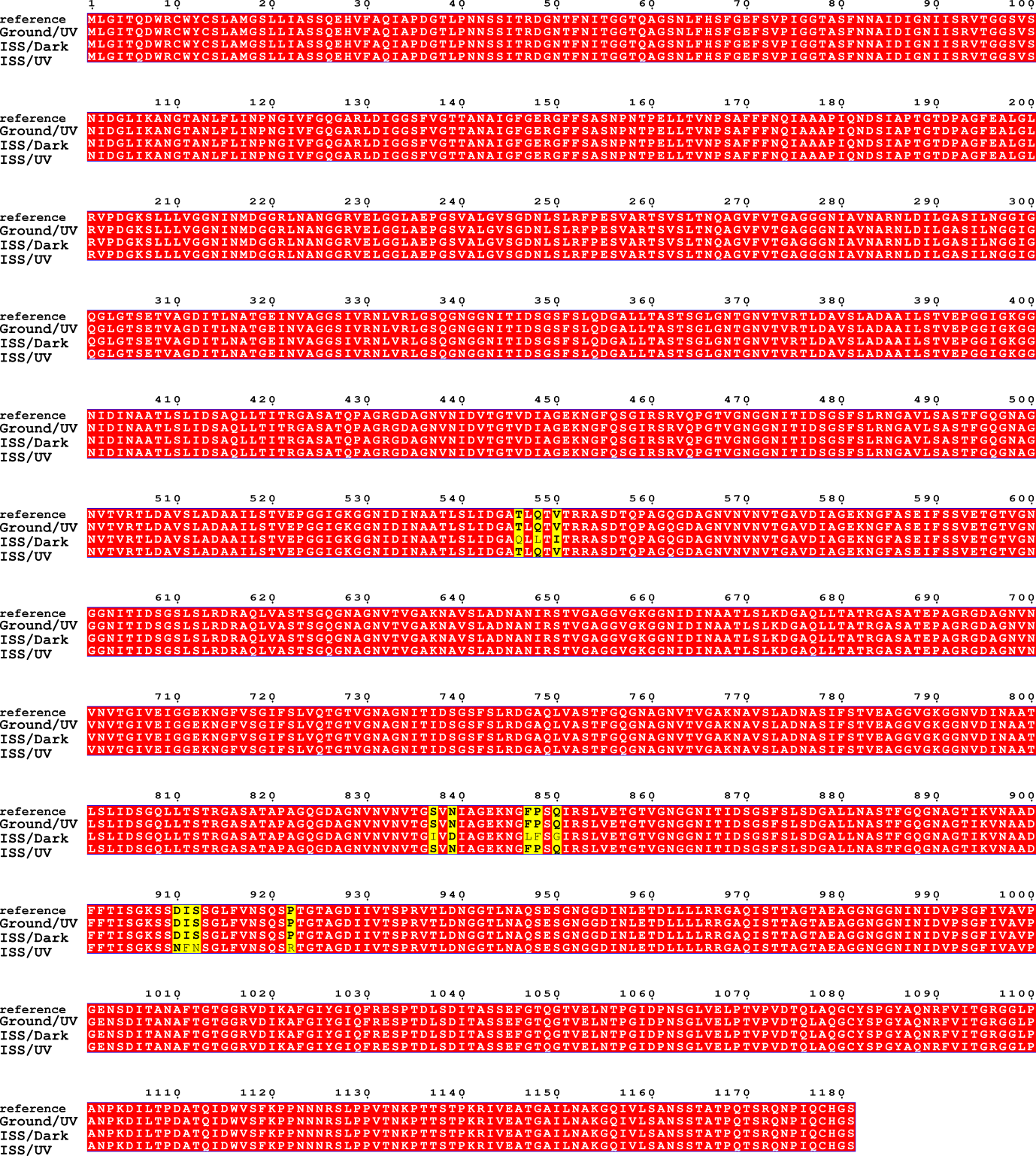


**Figure S4.** Multiple sequence alignment of a putative hemagglutinin-related protein (CCCRYO23106-428:42166-45711), for all three experimental conditions and the consensus amino acid sequence reference. ISS – International Space Station; UV – Ultraviolet Light.


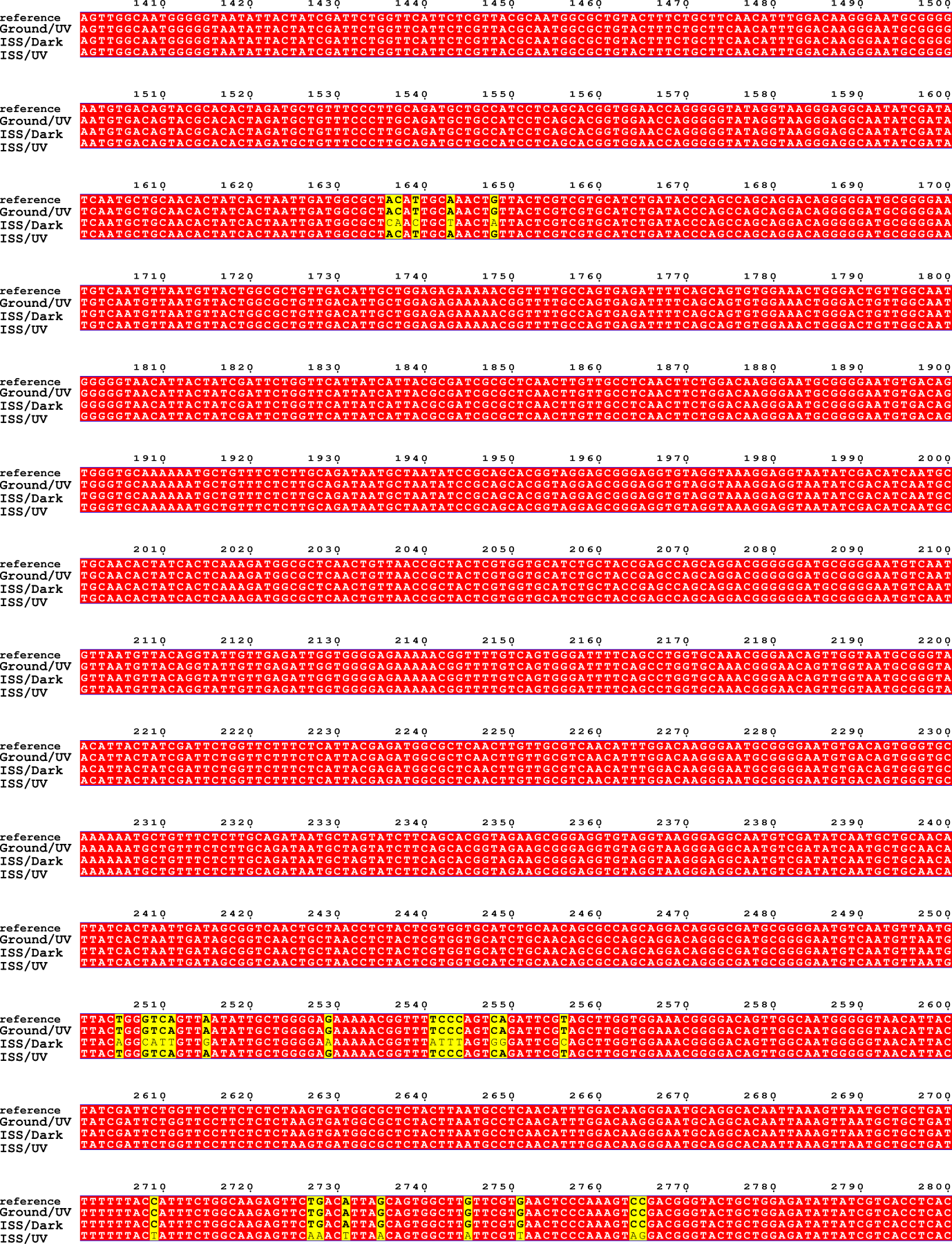


**Figure S5.** Multiple sequence alignment of the selected nucleotide sequences of a putative hemagglutinin-related gene (CCCRYO23106-428:42166-45711) displaying all variants seen in Figure S6, for the three experimental conditions and the consensus sequence reference, showing only a few additional synonymous variants. ISS – International Space Station; UV- Ultraviolet Light.


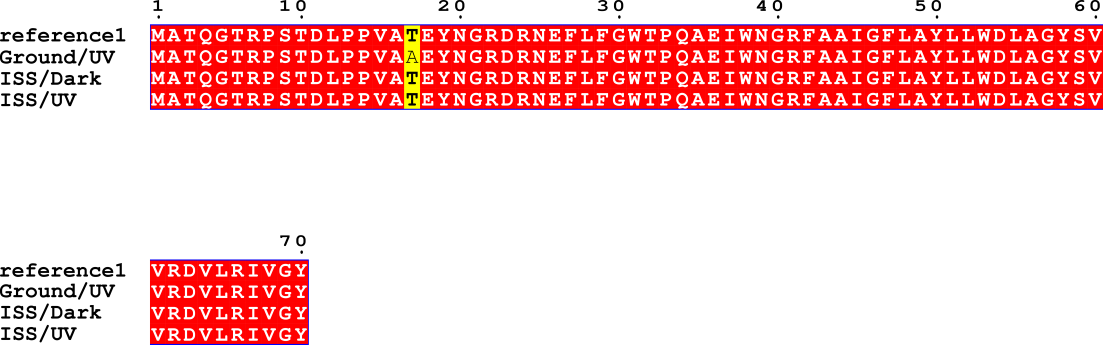


**Figure S6.** Multiple sequence alignment of a High Light Inducible Protein (CCCRYO23106-546:4255-4467), for all three experimental conditions and the consensus amino acid sequence reference. ISS – International Space Station; UV- Ultraviolet Light.


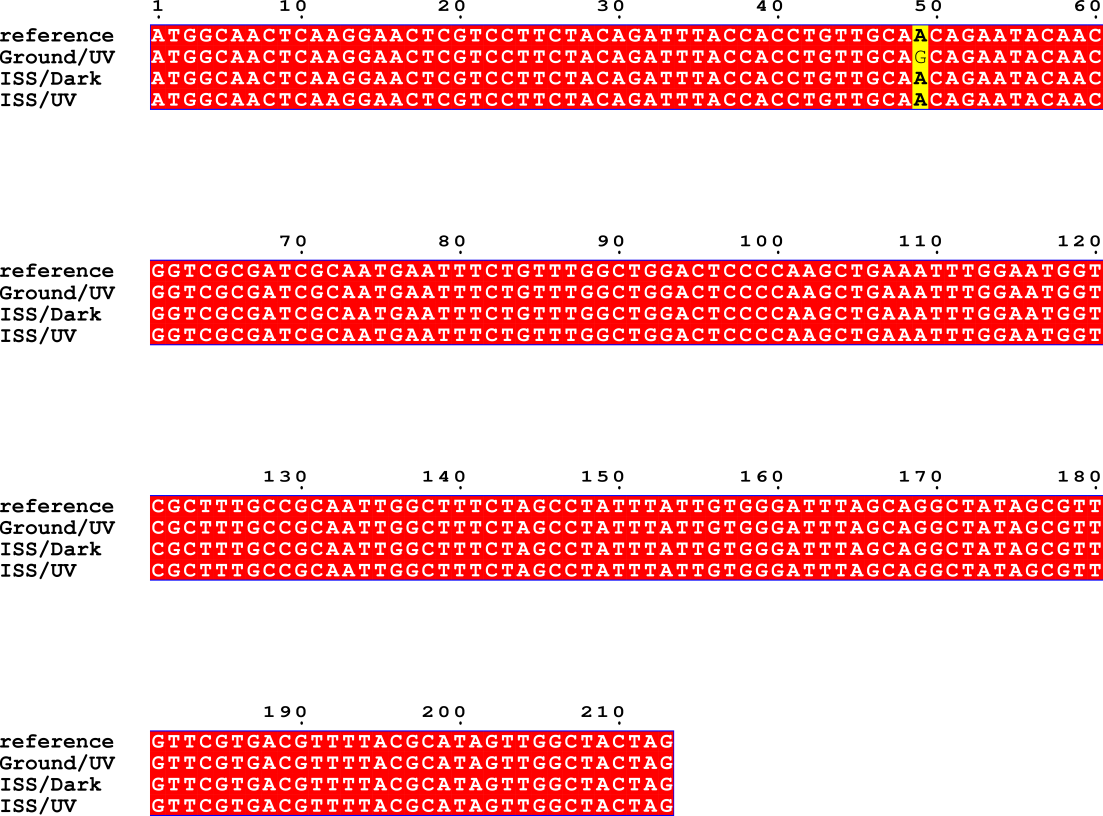


**Figure S7.** Multiple sequence alignment of the nucleotide sequences of a High Light Inducible Protein-family gene (CCCRYO23106-546:4255-4467), for all three experimental conditions and the consensus amino acid sequence reference. Only a single variant is detected, which results in a non-synonymous change in the amino acid sequence. ISS – International Space Station; UV- Ultraviolet Light.


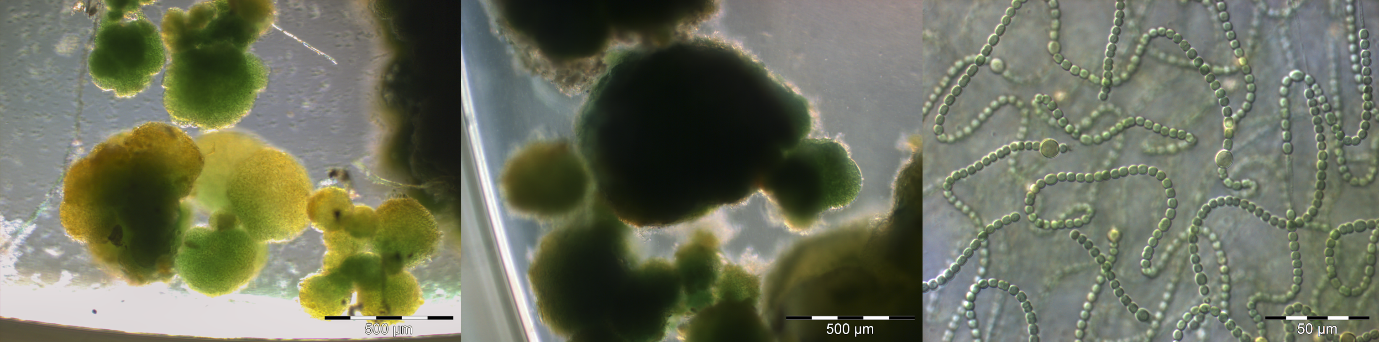


**Figure S8.** Rehydration and growth of *Nostoc* sp. strain CCCryo 231-06 after return from the International Space Station (ISS). Left: 3 days after the Sulfatic Martian Regolith (S-MRS/Dark) samples that returned from the ISS were inoculated with fresh culture medium. *Nostoc* looked intensely pigmented after rehydration and showed regrowing with some petrol-colored colonies. Center: 3 days after the Phyllosilicatic Martian Regolith (P-MRS/Dark) samples that returned from the ISS were inoculated with fresh culture medium. Petrol-colored colonies observed. Right: 23 months after return of the samples from the ISS. Heterocysts capable of fixing atmospheric nitrogen can be observed (larger and slightly yellow cells in filament).

**References**

1. Rabbow E, Rettberg P, Parpart A, Panitz C, Schulte W, Molter F, et al. EXPOSE-R2: the astrobiological ESA mission on board of the International Space Station. Frontiers in Microbiology. 2017;8:1533.

2. Dachev TP, Bankov N, Tomov B, Matviichuk YN, Dimitrov PG, Häder DP, et al. Overview of the ISS Radiation Environment Observed during the ESA EXPOSE‐R2 Mission in 2014–2016. Space Weather. 2017;15(11):1475-89.

3. De Vera J-P, Alawi M, Backhaus T, Baqué M, Billi D, Böttger U, et al. Limits of life and the habitability of Mars: the ESA space experiment BIOMEX on the ISS. Astrobiology. 2019;19(2):145-57.

4. Li H, Wysoker A. Durbin R; 1000 Genome project data processing subgroup. The sequence alignment/Map format and SAMtools. 2009.

5. Didion JP, Martin M, Collins FS. Atropos: specific, sensitive, and speedy trimming of sequencing reads. PeerJ. 2017;5:e3720.

6. Chu J, Sadeghi S, Raymond A, Jackman SD, Nip KM, Mar R, et al. BioBloom tools: fast, accurate and memory-efficient host species sequence screening using bloom filters. Bioinformatics. 2014;30(23):3402-4.

7. Wood DE, Lu J, Langmead B. Improved metagenomic analysis with Kraken 2. Genome biology. 2019;20(1):257.

8. Pirooznia M, Kramer M, Parla J, Goes FS, Potash JB, McCombie WR, et al. Validation and assessment of variant calling pipelines for next-generation sequencing. Human genomics. 2014;8(1):14.

9. Robert X, Gouet P. Deciphering key features in protein structures with the new ENDscript server. Nucleic acids research. 2014;42(W1):W320-W4.
